# Serpin Signatures in Prion and Alzheimer’s Diseases

**DOI:** 10.1007/s12035-022-02817-3

**Published:** 2022-04-13

**Authors:** Marco Zattoni, Marika Mearelli, Silvia Vanni, Arianna Colini Baldeschi, Thanh Hoa Tran, Chiara Ferracin, Marcella Catania, Fabio Moda, Giuseppe Di Fede, Giorgio Giaccone, Fabrizio Tagliavini, Gianluigi Zanusso, James W. Ironside, Isidre Ferrer, Giuseppe Legname

**Affiliations:** 1 Laboratory of Prion Biology, Department of Neuroscience, Scuola Internazionale Superiore Di Studi Avanzati (SISSA), Trieste, Italy; 2 Present Address: German Center for Neurodegenerative Diseases (DZNE), 72076 Tübingen, Germany; 3 Present Address: Osteoncology Unit, Bioscience Laboratory, IRCCS Istituto Romagnolo Per Lo Studio Dei Tumori (IRST) “Dino Amadori”, 47014 Meldola, Italy; 4 Present Address: Institute of Biomedicine, Department of Pathology and Experimental Therapeutics, Bellvitge University Hospital-IDIBELL, Barcelona, Spain; 5 Present Address: VN-UK Institute for Research and Executive Education, The University of Danang, Da Nang, Vietnam; 6 Division of Neurology 5 and Neuropathology, Fondazione IRCCS Istituto Neurologico Carlo Besta, Milan, Italy; 7 Scientific Directorate, Fondazione IRCCS Istituto Neurologico Carlo Besta, Milan, Italy; 8 Department of Neurosciences, Biomedicine and Movement Sciences, University of Verona, Verona, Italy; 9 National CJD Research & Surveillance Unit, Centre for Clinical Brain Sciences, University of Edinburgh, Edinburgh, UK; 10 Department of Pathology and Experimental Therapeutics, University of Barcelona, Hospitalet de Llobregat, Spain; 11 Institute of Biomedical Research of Bellvitge (IDIBELL), Hospitalet de Llobregat, Spain; 12 Biomedical Research Network Center of Neurodegenerative Diseases (CIBERNED), Hospitalet de Llobregat, Spain

**Keywords:** Prion diseases, Alzheimer’s disease, Gene expression, SERPINA3/SerpinA3n

## Abstract

Serpins represent the most broadly distributed superfamily of proteases inhibitors. They contribute to a variety of physiological functions and any alteration of the serpin-protease equilibrium can lead to severe consequences. SERPINA3 dysregulation has been associated with Alzheimer’s disease (AD) and prion diseases. In this study, we investigated the differential expression of serpin superfamily members in neurodegenerative diseases. *SERPIN* expression was analyzed in human frontal cortex samples from cases of sporadic Creutzfeldt-Jakob disease (sCJD), patients at early stages of AD–related pathology, and age-matched controls not affected by neurodegenerative disorders. In addition, we studied whether *Serpin* expression was dysregulated in two animal models of prion disease and AD.

Our analysis revealed that, besides the already observed upregulation of *SERPINA3* in patients with prion disease and AD, *SERPINB1*, *SERPINB6*, *SERPING1*, *SERPINH1*, and *SERPINI1* were dysregulated in sCJD individuals compared to controls, while only *SERPINB1* was upregulated in AD patients. Furthermore, we analyzed whether other serpin members were differentially expressed in prion-infected mice compared to controls and, together with *SerpinA3n*, *SerpinF2* increased levels were observed. Interestingly, SerpinA3n transcript and protein were upregulated in a mouse model of AD. The SERPINA3/SerpinA3nincreased anti-protease activity found in post-mortem brain tissue of AD and prion disease samples suggest its involvement in the neurodegenerative processes. A SERPINA3/SerpinA3n role in neurodegenerative disease-related protein aggregation was further corroborated by *in vitro* SerpinA3n-dependent prion accumulation changes. Our results indicate SERPINA3/SerpinA3n is a potential therapeutic target for the treatment of prion and prion-like neurodegenerative diseases.

**Supplementary Information:**

The online version contains supplementary material available at 10.1007/s12035-022-02817-3.

## Introduction

Serpins represent the largest and most widely distributed superfamily of protease inhibitors [1], which are found in all three domains of life [2–4]. Eukaryotic serpins have been divided into sixteen clades according to their sequence similarity [5, 6].

The human serpin superfamily accounts for thirty-six members and five pseudogenes represented by the first nine clades (from A to I) with the largest part of them playing mainly an inhibitory role [7].

Mouse serpins account for sixty functional genes, the majority of which are orthologous of human serpins while some have been expanded into multiple paralogue genes [7, 8].

Sequence homology analysis has revealed that members of the serpin family are phylogenetically grouped by species and not by function. This categorization highlights the hypothesis that the evolution of this family probably did not occur in parallel with serine proteases, but instead it may be due to speciation to fulfill their role in different biological processes [9].

Human SERPINA3 (also known as α1-antichymotrypsin) is a glycoprotein belonging to the serine protease inhibitor family of acute phase proteins [10]. A gene located in a cluster on chromosome 14q32.1, including nine other serpins, encodes for SERPINA3.

As for the human gene, murine clade A3 serpin is present in a cluster of fourteen genes (named SerpinA3a-n) located on chromosome 12F1. Importantly, SerpinA3n shares 70% homology with the human gene [11]; therefore, it has been considered as the functional orthologous of human SERPINA3 in the brain [12]

The expansion of SERPINA3 gene is not restricted to mouse species, indeed there are also six antichymotrypsin-like serpins in the rat genome. Therefore, the presence of only one α_1_-antichymotrypsin gene in humans, in contrast to the mouse and rat, seems to be an exception rather than a rule. This may be due to gene loss, even if further studies on the clade A cluster in other primates should be carried out to confirm this hypothesis [11, 12].

As for the human SERPINA3, its murine orthologue also shows a wide tissue distribution, being expressed in the liver, brain, testis, lungs, thymus, and spleen, and to a lesser extent in the bone marrow, skeletal muscle, and kidney [13].

In the central nervous system (CNS), the primary source of SERPINA3 is astrocytes, where its expression is upregulated by IL-1, TNF, oncostatin M, IL-6 soluble, and IL-6 receptor complexes [14–16].

Both SERPINA3 and SerpinA3n are involved in the same physiological processes such as the complement cascade, apoptosis, wound healing, inflammation, and extracellular matrix remodeling. Furthermore, together with their biological roles, they were found to be overexpressed in various different pathologies [8].

Physiologically, the basal level of SERPINA3 expression in the brain is very low, but immunohistochemical analysis reveals the presence of SERPINA3 in activated astrocytes during aging, both in human and monkeys [17, 18]. Upregulation of SERPINA3 in the brain of non‐demented people above age 65 compared to younger individuals was also observed, further confirming its involvement in aging process [19].

In 2014, a microarray-based gene expression study revealed the upregulation of SERPINA3 transcript in brains from bovine spongiform encephalopathy (BSE)–infected cynomolgus macaques, which are considered a highly relevant model for variant Creutzfeldt-Jakob disease (vCJD) [20]. SERPINA3 upregulation has already been observed in the CNS of sporadic Creutzfeldt-Jakob disease (sCJD) patients, while cerebrospinal fluid (CSF) and urine samples from these patients also revealed high level of SERPINA3 protein [21]. Western blot (WB) and RT-qPCR analysis of human frontal cortex specimens from patients affected by other types of prion diseases confirmed elevated levels of SERPINA3 [22].

Moreover, increased level of *SerpinA3n* mRNA has been found in different mouse prion disease models [23–28], where its expression progressively increases during the course of the disease [22, 29, 30]

Prion diseases, also known as transmissible spongiform encephalopathies (TSEs), are a class of fatal neurodegenerative diseases affecting both human and animals [31, 32]. The etiological agents responsible for TSEs are prions, which consist of an abnormally folded protein (known as PrP^Sc^) that accumulates in CNS either in the form of plaques or as synaptic deposits [31, 32]. Prions are able to aggregate and propagate by acting as corruptive templates (seeds) for the pathological conversion of the physiological cellular prion protein, PrP^C^, normally expressed in the cell membrane of CNS neurons, into PrP^Sc^ [31, 33]. sCJD is the most common type of human prion disease and accounts for more than 80% of all cases, with an incidence of about 1.5 cases per million [34]. Prion diseases can be caused by the spontaneous conversion of PrP^C^ into PrP^Sc^ (as proposed for sCJD) or by somatic mutations in the *PRNP* gene in familial forms of CJD, which render PrP^C^ susceptible to misfolding and aggregation.

At the molecular level, other neurodegenerative diseases such as Alzheimer’s disease (AD) and Parkinson’s disease (PD) share a similar pathogenic mechanism of seeded aggregation of disease-specific proteins. The misfolded protein induces a conformational change of the physiological protein, leading to the formation of other proteopathic seeds able to spread among different brain regions [35–38]. Recently, it has been proposed that this mechanism also characterizes Amyloid-β (Aβ), tau, α-synuclein, SOD1, TDP-43, and huntingtin, involved in AD, PD, amyotrophic lateral sclerosis (ALS), frontotemporal lobar degeneration, and Huntington’s disease, respectively.

AD is the most common neurodegenerative disorder in elderly, where 95% of cases are sporadic [39]. Amyloid plaques consisting of the extracellular accumulation of abnormally folded Aβ with 40 or 42 amino acids (Aβ40 and Aβ42), and intracellular neurofibrillary tangles (NFT), mainly composed of paired helical filaments of hyperphosphorylated tau protein, represent the two main neuropathological hallmarks of AD.

Concerning the prion-like mechanism of AD, it has been shown that the intracerebral injection of AD brain homogenates into marmosets produces the appearance of senile plaques accompanied by dystrophic neurites and cerebral amyloid angiopathy, in the absence of NFT [40]. Thanks to the development of Aβ precursor protein (APP) transgenic mouse models, different studies have highlighted the possibility to recapitulate the disease through the injection of Aβ-enriched brain extracts, thus confirming Aβ prion–like behavior [41–43].

SERPINA3 was firstly linked to AD when it was found to be a relevant component of amyloid brain deposit, being highly expressed in AD–affected brain regions [18]. SERPINA3 overexpression appears to be involved in the progression of AD [44–46] and, in line with this hypothesis, we previously observed upregulation of *SERPINA3* transcript in human frontal cortex samples of patients at early stages of AD–related pathology [22]. Furthermore, a high concentration of SERPINA3/SerpinA3n has been found both in the CSF and brain of AD patients and AD animal models, respectively [47, 48].

In situ hybridization studies on AD brains have shown that SERPINA3 is mainly produced by reactive astrocytes surrounding senile plaques. This is also confirmed by a high level of the corresponding soluble protein in this cell type and by RNA-sequencing analysis of AD brains [49, 50]. However, transcriptomic and immunofluorescence analyses of brains from 5XFAD mice undergoing Aβ accumulation revealed the appearance of SerpinA3n positive oligodendrocyte cell populations, which provide some evidence for a difference in the cell type of origin between mice and humans [50].

Interestingly, a SERPINA3 signal peptide polymorphism at codon 17 (A/T), in combination with APOE4 allele, has been associated with an increased susceptibility to AD [51]. Furthermore, the correlation between APOE4 and SERPINA3/SerpinA3n has been recently confirmed, from the observation that human APOE4-targeted replacement in mice leads to increased expression of SERPINA3 family genes in their brains [52]. This finding was also supported by the co-localization of APOE and SERPINA3/SerpinA3n found in amyloid plaques [47].

Interestingly, SERPINA3 polymorphisms are associated with an increased susceptibility to neurological illnesses and may be related to early onset of PD and Multiple System Atrophy (MSA) [53, 54]. SERPINA3 was also found to be significantly upregulated in the motor cortex of ALS patients [55] and expressed four-fold more in the MSA frontal cortex compared to controls [56], suggesting SERPINA3 involvement in other types of neurodegenerative diseases.

In this study, we address whether other members of the serpin superfamily are differentially expressed in the frontal cortex of patients affected by sCJD and at early stages of AD–related pathology, in comparison to non-neurodegeneration-affected controls.

Considering the *SerpinA3n* upregulation observed in prion-infected CD1 mouse brain [22], we also analyzed the levels of other mouse serpins in infected mice. In addition, differential expression of serpins was also addressed in an AD mouse model, huAPP^Swe^/moAPP^0/0^, to better elucidate a possible correlation between serpin expression and prion-like pathologies.

Furthermore, we tested the anti-protease activity of the most upregulated serpin, SERPINA3/SerpinA3n, in AD– and prion-affected brain tissue. Finally, we assessed whether *SerpinA3n* modulation would affect prion accumulation in in vitro model of the disease.

## Materials and Methods

### Patient Samples

A total of 45 frontal cortex tissue samples from neurodegeneration-affected patients and control subjects were collected. The study was performed on samples coming from the following cases: early stages of NFT pathology, Braak stages I–III (referred herein as AD (*n* = 15)) and sCJD (*n* = 15). Age-matched subjects who had died from unrelated conditions, lacking any neurological signs in life or pathological lesions in brain, were included as controls (*n* = 15). Cases diagnosed as sCJD were all confirmed by means of neuropathological analysis and the detection of PrP^Sc^ by WB, while AD diagnoses were confirmed through neuropathological analysis. Tissue samples and associated data were provided by the MRC Edinburgh Brain Bank (UK), the Institute of Neuropathology Brain Bank (HUB-ICO-IDIBELL Biobank) (Barcelona, Spain), the Carlo Besta Neurological Institute (Milan, Italy), and the University hospital of Verona (Italy). The full list of samples and patient details is reported in Table 1. An additional control sample (1615, 83 years old female) from University hospital of Verona (Italy) was included for the analysis of SERPINA3 activity in brain tissue.

**Table 1:**
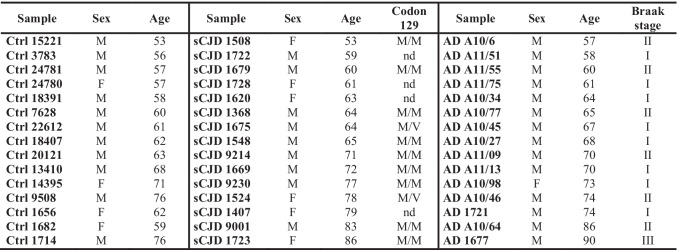
Human brain samples for RT-qPCR analysis. Sex and age of non-neurodegeneration-affected controls (CTRLs), sporadic Creutfeldt-Jakob (sCJD), and Alzheimer’s disese (AD) cases are included in the present study. The status of the codon 129 methionine/valine polymorphism in the human prion proteine gene is recorded (where available) for sCJD cases. *F* female, *M* male, *MM* methionine/methionine, *MV* methionine/valine, *VV* valine/valine

### Mouse Samples

Rocky Mountain Laboratory (RML)–infected CD1 mice brain samples both at pre-symptomatic (3 months post injection, *n* = 3) and symptomatic stage of the diseases (5 months post injection, *n* = 4) and related age- and sex-matched controls (5 months of age, *n* = 3 and 7 months of age, *n* = 4) were provided by Dr. Moda (Fondazione IRCCS Istituto Neurologico Carlo Besta, via Celoria 11, Milan, Italy).

Transgenic APP23 mice expressing the Swedish double mutation in the human APP gene (K595N/M596L) and knock-out for endogenous APP were provided by Prof. Di Fede (Fondazione IRCCS Istituto Neurologico Carlo Besta, via Celoria 11, Milan, Italy). They were generated by crossing APP23 mice with moAPP^0/0^ animals and sacrificed at 12 months (moAPP^0/0^/huAPP^Swe^, *n* = 9). Mice knock-out for the endogenous APP were used as control (moAPP^0/0^, *n* = 9).

### Data Sources

Selection of serpins for gene expression analysis was performed using different databases. To evaluate the level of expression of *SERPIN* genes in human brain tissue, Consensus Normalized Expression dataset was used (https://www.proteinatlas.org/). It combines data from the three transcriptomic datasets: HPA, GTEx, and FANTOM5.

For mouse serpin genes, two different databases were used. Data were analyzed from Mouse ENCODE transcriptome (https://www.ncbi.nlm.nih.gov/bioproject/PRJNA66167/) and Allen brain Atlas (https://mouse.brain-map.org/).

For human genes, we set a threshold value of Transcripts Per Kilobase Million (TPM) ≥ 2 [57] while a cut-off value of Reads Per Kilobase Million (RPKM) ≥ 1 was used for mouse genes [58]. Following this analysis, twelve and seventeen serpin transcripts were chosen for human and mouse, respectively.

### RNA Extraction

Total RNA from human samples and prion-infected mouse brains was isolated as already described in Vanni et al. (2017). For huAPP^Swe^/moAPP^0/0^ and moAPP^0/0^ mice, one brain hemisphere was homogenized using T 10 basic ULTRA-TURRAX® homogenizer (IKA Dispersers) in 1 mL TRIzol reagent. RNA was extracted with PureLink® RNA Mini Kit (Life Technologies) and on-column DNA digestion was performed using PureLink DNase Set (Life Technologies). RNA was checked for concentration and purity on a NanoDrop 2000 spectrophotometer (Thermo Scientific), whereas RNA integrity was analyzed using 2100 Bioanalyzer (Agilent Technologies).

### Reverse Transcription Quantitative Polymerase Chain Reaction (RT-qPCR)

For human frontal cortex, mouse brain, and cell line samples, cDNA was obtained starting from 3 µg of total RNA as already described in Vanni et al. (2017). A negative control was performed for each sample by omitting the reverse transcriptase.

qPCR primers were designed using the outline tool Primer-Blast provided by NCBI and validated using the online tool OligoCalc (http://biotools.nubic.northwestern.edu/OligoCalc.html). Alternatively, qPCR human primer for *SERPINB6* was taken from [59]; *SERPINB9* from [60]; *SERPINF1* from [61]; *SERPING1* from [62]; and *SERPINH1* from [63]. For what concerns qPCR mouse primer, *SerpinD1* and *SerpinF2* were taken from [64], *SerpinE2* from [65], and *Serpici1* from [66] (Online Resource 1–2).

Gene expression assays were performed using iQ™ SYBR® Green Supermix (Bio-Rad Laboratories, Inc.) with CFX96 Touch™ Real-Time PCR Detection System (Bio-Rad Laboratories, Inc.) as previously described. qPCR reaction was performed in 96-well plates, in duplicates for each primer pair and sample.

### RT-qPCR Data Analysis

Differential expression of target human genes was normalized to three different reference genes (*ACTB*, *GAPDH*, and *B2M*) expression. The expression stability of these three housekeeping genes was previously assessed in both disease and control patients and they effectively showed comparable expression levels among the different groups [67]. For mouse samples, *Actb*, *Gapdh*, and *Tubb3* were used as reference genes.

The absolute expression value (C_T_) of each serpin gene was addressed in human/mouse brain pool to select genes having a C_T_ ≤ 35 [22]. RT‐qPCR analysis for selected serpin mRNA was performed in the frontal cortex of 45 human brains and whole brain of fourteen CD1 mice and eighteen APP23 mice. The relative gene expression ratio (fold change, FC) was calculated using 2^−ΔΔCT^ method [68] as reported in Vanni et al. 2018 [67].

ΔC_T_ was calculated subtracting the C_T_ of the housekeeping gene to the C_T_ of the target one, both for “test” (disease-affected patient/infected mouse) and “calibrator” (control). Then, ΔΔC_T_ was obtained subtracting the mean ∆C_T_ of the population of calibrator samples (fifteen samples for human analysis; three and four samples for the pre- and symptomatic stages of prion-infected mice analysis, respectively; and nine samples for AD mouse model analysis) from the ∆C_T_ of each sample (both of calibrator and test). Fold change (FC) values smaller than 1 were converted using the equation − 1/FC, for representation.

### Cell Culture

N2a and RML chronically infected N2a cell lines (ScN2a RML) were grown in Minimal Essential Medium (MEM)-1X, GlutaMAXTM supplement (Gibco, Thermo Fisher Scientific), supplemented with 10% fetal bovine serum (FBS), 1% non-essential amino acids, and 1% penicillin–streptomycin. All cell lines were cultivated in 10 cm^2^ or 6 cm^2^ Petri dishes (Falcon) at 37 °C under 5% CO_2_.

### N2a Overexpressing SerpinA3n

*Serpina3n* coding sequences were amplified from mouse cDNA using the following primers: 5’- GGATATCTGCAGAATTCATCATGGCCTTCATTGCAGCTCTGGGG-3’, 5’- GCTTGGTACCGAGCTCGGATCCTCATTTGGGGTTGGCTATCTTGGC-3’. *SerpinA3n* gene was cloned to pcDNA3.1 vector by restriction-free cloning, resulting in the recombinant plasmid pcDNA3.1/*SerpinA3n*. Briefly, 0.4 µg DNA was diluted with Buffer EC (Qiagen), to a total volume of 100 µL. A total of 3.2 µL enhancer was added and mixed. Following a 5-min incubation at room temperature (RT), 10 µL Effectene Transfection Reagent (Qiagen) was added to the DNA-Enhancer mixture and incubated for a further 10 min at RT. DNA transfection mixture was added to the cells in a dropwise manner. Cells were incubated with DNA transfection mixture for 18 h at 37 °C, following which FBS–supplemented OPTI-MEM medium was replaced. Forty-eight hours after transfection, cells were split into fresh medium containing 1 mg/mL Geneticin (Gibco). The selective medium was changed every 3–4 days until Geneticin-resistant foci could be identified. After that, the stable cell lines were maintained in medium containing 400 µg/mL Geneticin. Transfection with empty pcDNA 3.1 plasmid (empty vector, EV) was performed in N2a as control (N2a-EV).

### Recombinant SerpinA3n Production

SepinA3n recombinant protein was produced as already described [69], with some modifications. A pET(11a) expression vector containing the C-terminally (6x) His-tagged murine SerpinA3n (Genetech) was used to transform *E. Coli* BL21 (DE3) pLysS cells. Cells were grown in Luria–Bertani medium at 25 °C in presence of ampicillin (100 µg/mL) until OD_600_ = 1, and then protein production was induced with 0.1-mM isopropyl 1-thio-D-galactopyranoside for 21 h at 15 °C. Bacterial cell lysis was performed by sonication using 5 cycles of 60 s on and 60 s of rest in ice. Cell debris was discarded by centrifugation and the crude extract was collected as the supernatant. The recombinant SerpinA3n protein was purified using HisTrap™ Fast Flow Crude column (GE Healthcare) with ÄKTA pure system. After eluting with a linear imidazole gradient, the fractions containing purified SerpinA3n were pooled together, dialyzed in 10 mM Tris–HCl and 50 mM KCl pH 8.0, and concentrated using Amicon® Ultra-15 Centrifugal Filters with 30 kDa cutoff (Merk Millipore).

### Conditioned Medium and Recombinant SerpinA3n Treatment

N2a-SerpinA3n*n*, N2a-EV, and N2a cells at 90% confluency were washed with phosphate-buffered saline (PBS) and fresh, FBS–depleted, Opti-MEM was added. Twenty-four hours later, conditioned medium (CM) was collected and cells were counted. 1 × 10^5^ ScN2a RML cells were plated in a volume of 3 mL MEM in a 6-cm^2^ plate (Falcon) 24 h before treatment. The following day, CM media from 1 × 10^6^ N2a-SerpinA3n, N2a-EV, and N2a cells were added to the cells and Opti-MEM was added to reach a final volume of 3 mL. For recombinant SerpinA3n treatment, 0.5 µM and 1 µM have been selected as suitable concentration. Vehicle-treated cells were treated with 10 mM Tris–HCl, 50 mM KCl pH 8.0. Seventy-two hours after treatment, cells were washed with PBS and lysed on ice in lysis buffer (10 mM Tris–HCl pH 8.0, 150 mM NaCl, 0.5% nonidet P-40, 0.5% deoxycholic acid sodium salt). Nuclei and large debris were removed with a centrifugation at 2000 × g for 10 min at 4 °C. Protein concentration was determined by Bicinchoninic Acid (BCA) method.

### siRNA Transfection

MISSION® esiRNAs targeting *SerpinA3n* and *EGFP* (Sigma-Aldrich) were transfected on ScN2a RML. Transfection was performed using Lipofectamine 3000 (Invitrogen) according to the manufacturer’s guidelines. Briefly, 3.5 µL Lipofectamine 3000 and 121.5 µL of FBS–depleted Opti-MEM were vortex for 5 s. Then, 112.5 µL FBS–depleted Opti-Mem and 12.5 µL of siRNA (2.5 µg) were added to diluted Lipofectamine 3000 and incubated for 15 min at room temperature. Transfection mixture was added to the cells in a dropwise manner. Seventy-two hours later, cell lysate and medium were collected. Medium was cleared following Gueugneau et al. protocol [70]. Subsequently, it was concentrated 10X and proteins were subjected to an acetone precipitation step before BCA analysis.

### shRNA Production and Transfection

The selected *SerpinA3n* target sequence for shRNA construction was selected from position 508 to 529 (ACGGGTAGTGCCCTGTTTATT). The DNA duplex with overhang of *EcoRI* and *BamHI* was generated by annealing method using the following primers: sh850n-F: GATCCCCGGACGGGTAGTGCCCTGTTTATTCTCGAGAATAAACAGGGCACTACCCGTTTTTTTGAATG and Sh850n-R: AATTCATTCAAAAAAACGGGTAGTGCCCTGTTTATTCTCGAGAATAAACAGGGCACTACCCGTCCGG. Then, the resulting fragment was inserted in pU6-Luc-Zgreen plasmid at the site of *EcoRI* and *BamHI*, resulting in pU6-shSerpinA3n. A total of 1 µg of the DNA plasmid pU6-shSerpinA3n (shRNA-SerpinA3n) or pU6-Luc-Zgreen (shRNA-CTRL) was transiently transfected into ScN2a RML cells using Effectene Transfection Reagent. The cell lysates and medium were collected 72 h after transfection, as previously described.

### Human and Mouse Brain Sample Homogenates

Prion-infected mouse and human brain samples were homogenized in PBS 1X supplemented with proteinase inhibitor (cOmplete™ Protease Inhibitor Cocktail, Roche), at 10% w/v. APP23 mouse brain samples were homogenized in PBS 1X at 10% w/v. Samples were then centrifuge for 1 min at 4°C (800 × g). Protein concentration of brain homogenates was determined by BCA method.

### Complex Formation Assay

The ability of SerpinA3n to inhibit the serine protease α-chymotrypsin (Merk) was observed following Horvath et al. protocol [13]. A total of 1 or 5 µM of SerpinA3n was incubated with 1 µM of chymotrypsin in 10 mM Tris–HCl, 50 mM KCl, and pH 8.0. Similarly, CM from N2a-SerpinA3n was concentrated 20X using Amicon® Ultra-15 Centrifugal Filters 30 kDa cutoff (Merk Millipore) and 10 μL of the concentrate were incubated with 100 ng chymotrypsin in PBS 1X. Reactions were incubated at 37 °C for 30 min. To check SERPINA3/SerpinA3n activity in tissue, 100 µg of human (1615 and 1714 as controls, and A10/34 and A11/75 as AD patients) or mouse brain samples (5 and 7 months of age controls, and 3 and 5 months post RML-injection samples) was incubated at 37 °C for 15 min with 500 ng or 100 ng chymotrypsin, respectively. 5X SDS-PAGE loading buffer was added to the samples in a 1:5 ratio. They were denatured at 100 °C for 10 min and stored at − 20 °C until further processing or analysis.

### Western Blot

For secreted SerpinA3n protein detection, 50 µg of CM was added into 5X SDS-PAGE loading buffer in a 1:5 ratio. A total of 100 µg of APP23 brain homogenates was used for SerpinA3n and APP WB analysis. For PrP detection, cell lysates were split into two parts. One part (125 µg) was treated with 2.5 µg of PK (Roche) at 37 °C for 1 h. The reaction was arrested with 2 mM of phenylmethylsulphonyl fluoride (Sigma-Aldrich). The PK-digested samples were precipitated by centrifugation at 180,000 × g for 75 min at 4 °C in the OptimaTM MAX-XP Ultracentrifuge (Beckman Coulter) and the pellet was resuspended in 1X SDS- PAGE loading buffer. The non-PK-digested samples (15 µg) were added into 2X SDS-PAGE loading buffer in a 1:1 ratio. All samples were boiled for 10 min at 100 °C. The desired amount of protein was loaded onto 9% or 12% Acrylamide/Bis-acrylamide (Sigma-Aldrich) gels for APP and SerpinA3n or PrP detection, respectively, and separated by SDS-PAGE using SE 600 Ruby (GE Healthcare). Gels were then transferred to PVDF membrane (Millipore) using Criterion Blotter (Bio-Rad) for 2 h at 4 °C. Secreted SerpinA3n membranes were incubated for 2 min in shaking with Ponceau S solution (Sigma-Aldrich). After 1 h of blocking in 5% milk in TSB-T, membranes were incubated with 6E10 antibody (1:1000, Covance) for APP, polyclonal mouse SerpinA3n antibody (0.4 μg/mL, R&D Systems), human SERPINA3 antibody (1:500, Sigma-Aldrich), and W226 [71] (1 µg/mL, kindly provided by Prof. Krammerer) for PrP, overnight. Membranes were then incubated for 1 h with rabbit anti-goat HRP secondary antibody for murine SerpinA3n or goat anti-mouse HRP secondary antibody for W226 and 6E10 antibodies, or with goat anti-rabbit HRP for human SERPINA3 antibody. Subsequently, monoclonal anti-β-actin − peroxidase antibody (1:10,000, Sigma-Aldrich) was incubated for 1 h. Reactions were visualized by chemiluminescence on UVITEC using Immobilon Classico Western HRP substrate (Millipore). Densitometric analysis was carried out using UVIBand software (Cambridge).

### Statistical Analysis

The distribution of data was assessed by the D’Agostino-Pearson normality test. Differences between the ∆C_Ts_ of disease-affected and age-matched control group human samples were assessed using Kruskal–Wallis test. The level of significance was calculated using Dunn’s multiple comparisons test between ∆C_Ts_ of disease and the control group. Concerning mouse samples, the Mann–Whitney test was performed to address the level of significance. Statistical differences between N2a CM-, siRNA-, and shRNA-treated and untreated cells have been assessed with the Wilcoxon-matched pairs signed rank test. Statistical differences between recombinant SerpinA3n-treated and untreated cells were calculated with the Friedman test and the level of significance was calculated using Dunn’s multiple comparisons test. *p* values ≤ 0.05 were considered as statistically significant. All data were processed using GraphPad Prism 6.0.

## Results

### SERPIN Gene Expression Analysis in sCJD and AD–Affected Human Brains

Among 41 human *SERPIN* superfamily genes, twelve *SERPIN* transcripts (*SERPINA3, SERPINA8, SERPINB1, SERPINB6, SERPINB8, SERPINB9, SERPINE1, SERPINE2, SERPINF1, SERPINH1, SERPING1, SERPINI1*) met the established criteria of TPM ≥ 2 [57]; thus, they were selected for the transcriptomic analysis.

However, *SERPINB8*, *SERPINB9*, and *SERPINE1* were excluded from the analysis because their expression was not detected in our samples.

**Fig. 1:**
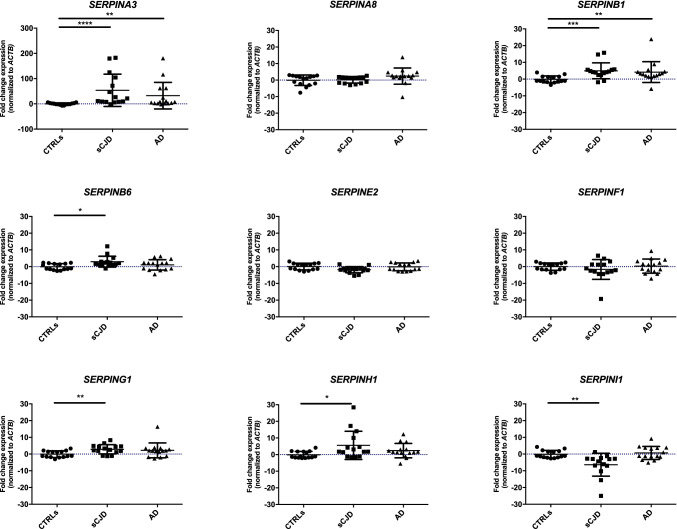
*SERPINs* expression level in sCJD and AD human brain samples normalized to *ACTB.* RT-qPCR for *SERPINs* mRNA expression in sCJD (*n* = 15) and AD (*n* = 15) relative age-matched controls (CTRLs, *n* = 15) frontal cortex samples normalized on *ACTB* as reference gene. Statistical analysis was performed using the Kruskal–Wallis test with Dunn’s multiple comparisons test. Adjusted *p* value * < 0.05, ** < 0.01, *** < 0.001, and **** < 0.0001

The results are shown using *ACTB* as reference gene (Fig. 1), although they showed similar trend when normalized using *GAPDH* and *B2M* (Online Resource 3–4).

Among the nine analyzed transcripts, as for the marked upregulation of *SERPINA3* in both sCJD (average FC = 54) and AD at early stages of NFT pathology (average FC = 32.7) groups [22], a significant upregulation of *SERPINB1* in affected patients (average FC = 5.2 for sCJD; average FC = 4.6 for AD) compared to controls was found. *SERPINI1*, instead, was significantly downregulated in sCJD (average FC =  − 3.1) group compared to age-matched controls, while no significant difference in the expression was found in AD at early stages of NFT pathology.

*SERPINB6*, *SERPING1*, and *SERPINH1* exhibited a significant upregulation in sCJD group (average FC = 3; average FC = 3.2; average FC = 6), whereas no differential expression was observed in AD group compared to controls.

No significant differences were found in *SERPINA8*, *SERPINE2*, and *SERPINF1* gene expression in patient groups compared to age-matched controls, also when normalized to the other reference genes (Online Resource 3–4).

### Serpin Gene Expression Analysis in Prion-Infected Mouse Brains

A threshold value of RPKM ≥ 1 was set [58] to select mouse serpin gene for the transcriptomic analysis. Seventeen transcripts among seventy-one mouse Serpins, *SerpinA3n*, *SerpinA6*, *SerpinA8*, *SerpinB1a*, *SerpinB1b*, *SerpinB6a*, *SerpinB8*, *SerpinB9d*, *SerpinD1*, *SerpinE1*, *SerpinE2*, *SerpinE3*, *SerpinF1*, *SerpinF2*, *SerpinG1*, *SerpinH1*, and *SerpinI1*, were selected and then analyzed by RT-qPCR. However, *SerpinA6*, *SerpinB9d*, and *SerpinE3* were not considered in our transcriptomic analysis because their expression was not detected in our samples.

**Fig. 2:**
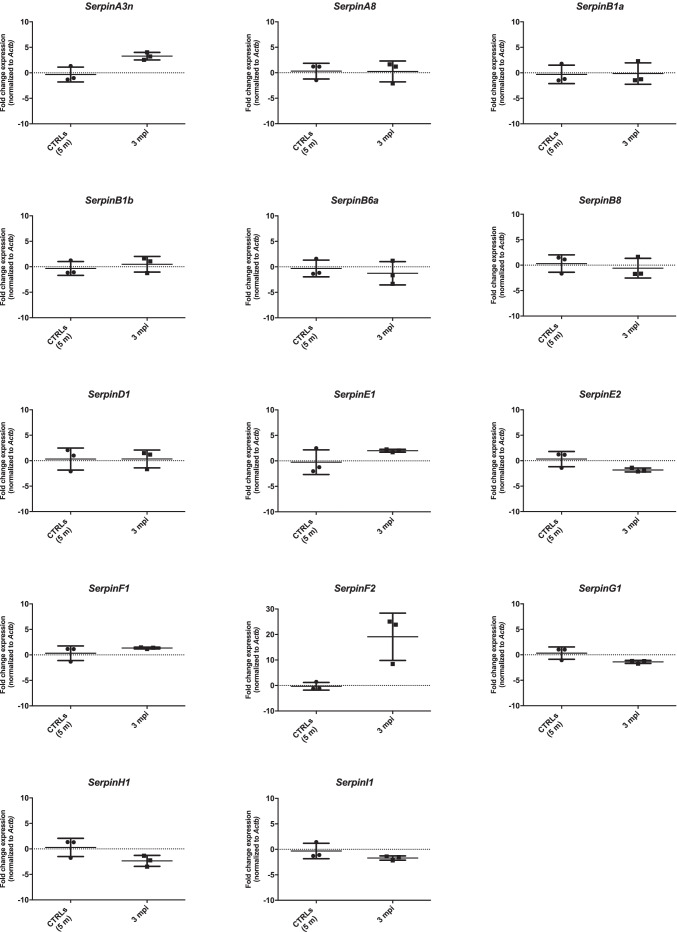
*Serpins* expression level in pre-symptomatic RML–infected CD1 mouse brain normalized to *Actb.* RT‐qPCR analysis for *Serpins* mRNA expression in 3 months post infection (3 mpi, *n* = 3) and relative age-matched control whole brain samples (CTRLs 5 m, *n* = 3) normalized to *Actb* as reference gene. Statistical analysis was performed with the Mann–Whitney test. Adjusted *p* value * < 0.05

Pre-symptomatic RML–infected CD1 mice (3 mpi) did not show any significant variation in *Serpin* expression compared to age-matched controls (5 mo). However, a trend of upregulation was present both for *SerpinA3n* and *SerpinF2* in 3 mpi mice (Fig. 2). The results were coherent for all the three housekeeping genes used for normalization (Online Resource 5–6).

**Fig. 3:**
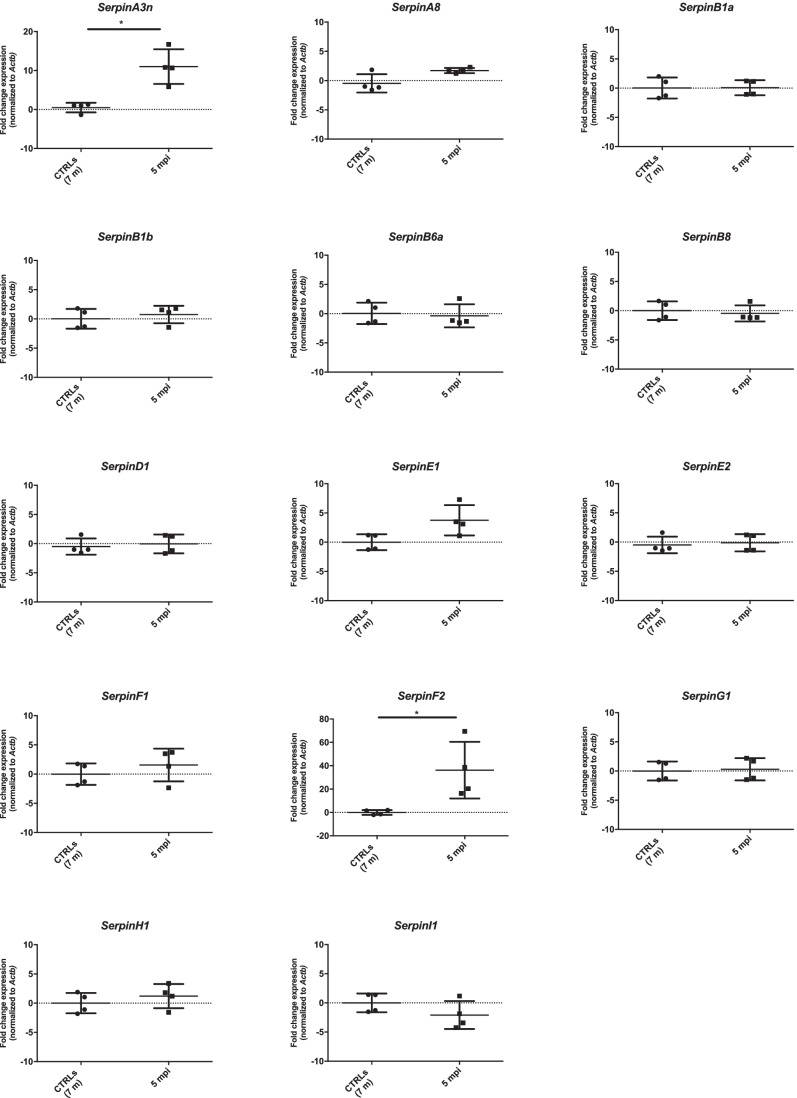
*Serpins* expression level in symptomatic RML–infected CD1 mouse brain normalized to *Actb.* RT‐qPCR analysis for *Serpins* mRNA expression in 3 months post infection (5 mpi, *n* = 4) and relative age-matched control whole brain samples (CTRLs 7 m, *n* = 4) normalized to *Actb* as reference gene. Statistical analysis was performed with the Mann–Whitney test. Adjusted *p* value * < 0.05

The majority of analyzed serpin transcripts did not show any significant variation in the expression between symptomatic RML–infected mice (5 mpi) and relative controls (7 mo) (Fig. 3). However, together with *SerpinA3n* (average FC = 11) [22], *SerpinF2* was significantly upregulated in prion-infected mice compared with control, with an average FC = 36. The results were shown using *Actb* as reference gene, but they were similar also when relative expression analysis was normalized with *Gapdh* and *Tubb3* (Online Resource 7–8).

### SerpinA3n Upregulation in Brain of AD Mouse Model

**Fig. 4:**
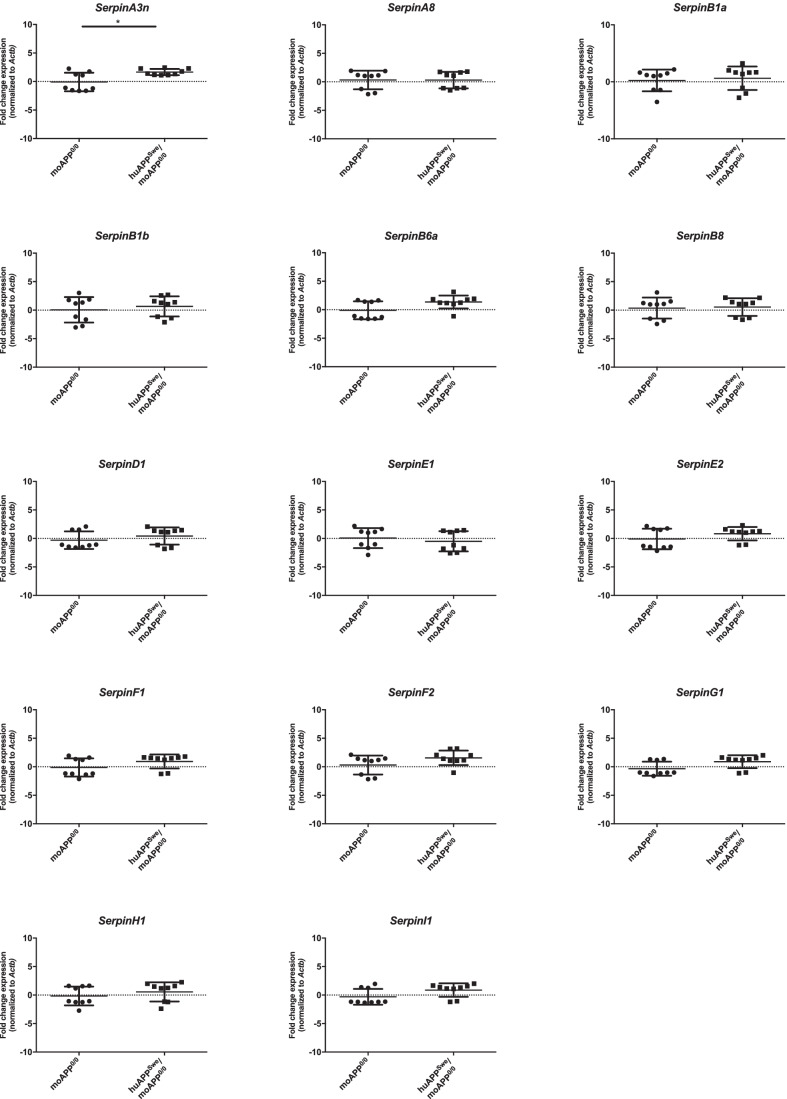
*Serpins* expression level in huAPP^Swe^/moAPP^0/0^ mouse brain normalized to *Actb.* RT‐qPCR analysis for *Serpins* mRNA expression in huAPP^Swe^/moAPP^0/0^ (*n* = 9) and relative age-matched control whole brain samples (moAPP^0/0^, *n* = 9) normalized to *Actb* as reference gene. Statistical analysis was performed with the Mann–Whitney test. Adjusted *p* value ** < 0.01

Using the same approach adopted for prion-infected mouse samples, serpin gene expression analysis was performed in moAPP^0/0^ and huAPP^Swe^/moAPP^0/0^ mouse brain. Among the analyzed transcripts, *SerpinA3n* (average FC = 2.3) was significantly upregulated in huAPP^Swe^/moAPP^0/0^ compared to moAPP^0/0^ both normalized versus *Actb* and *Gapdh* (Fig. 4 and Online Resource 9), while only a mild downregulation of *SerpinE1* in huAPP^Swe^/moAPP^0/0^ was observed when data were normalized on *Tubb3* (Online Resource 10).

**Fig. 5:**
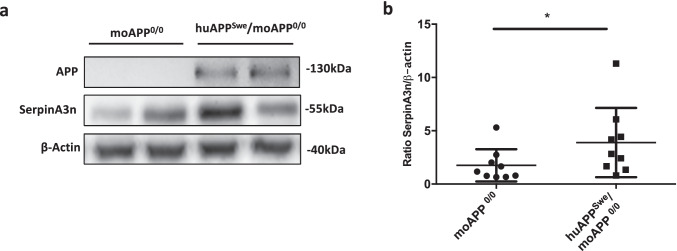
Western blotting analysis for SerpinA3n in the brain of moAPP^0/0^ and huAPP^Swe^/moAPP^0/0^ mouse. **a** Representative WB image of SerpinA3n protein level in moAPP^0/0^ and huAPP^Swe^/moAPP^0/0^ mouse brain lysates. β-actin was used as protein loading control and to normalize the expression level of SerpinA3n for densitometric analysis. **b** Densitometric analysis of SerpinA3n normalized on β-actin in moAPP^0/0^ and huAPP^Swe^/moAPP^0/0^ was shown. Statistical analysis was performed with the Mann–Whitney test. Adjusted *p* value * < 0.05

Since upregulation of SerpinA3n in RML–infected mice brain was found at both mRNA and protein level [22], WB for SerpinA3n detection was performed on moAPP^0/0^ and huAPP^Swe^/moAPP^0/0^ mouse brain homogenates. An upregulation of SerpinA3n protein in huAPP^Swe^/moAPP^0/0^ mouse brain relative to moAPP^0/0^ was observed (Online Resource 11). A representative image of WB and the related densitometric analysis were reported (Fig. 5a, b).

### SERPINA3/SerpinA3n Protease Inhibitor Activity in Prion and Alzheimer’s Disease Samples

**Fig. 6:**
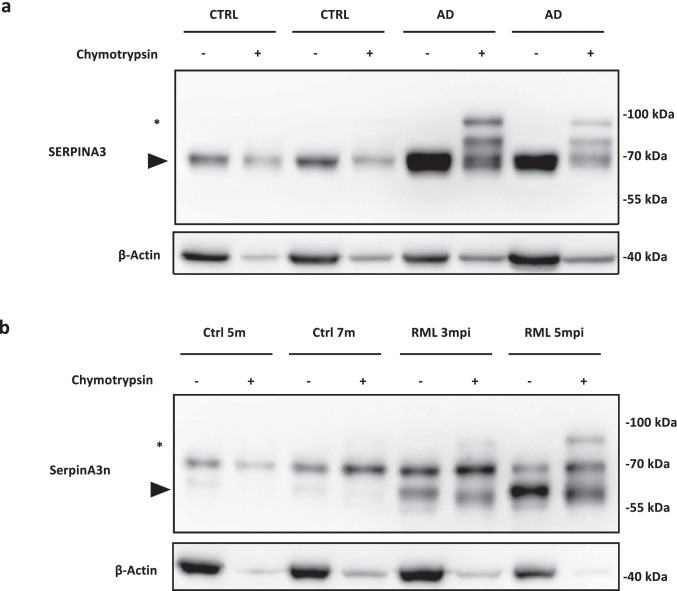
SERPINA3/SerpinA3n complex formation assay in AD and prion-affected samples. **a** WB analysis of SERPINA3 complex formation assay in AD at early stages of NFT pathology brain samples. Black arrowhead indicates signal of SERPINA3 and asterisk indicates SDS-stable SERPINA3-chymotrypsin complex. **b** WB analysis of SerpinA3n complex formation assay in RML–infected brain samples. Black arrowhead indicates signal of SerpinA3n and asterisk indicates SDS-stable SerpinA3n-chymotrypsin complex. β-actin was used as protein loading control

To test the protease inhibitor activity of upregulated SERPINA3/SerpnA3n found in AD and prion, we tested its ability to form an SDS-stable and covalent complex [72] together with chymotrypsin, one of its known target proteases [13]. WB analysis of frontal cortex samples from AD patients at early stages of NFT pathology revealed the presence of SERPINA3-chymotrypsin complex, which is consistent with the sum of the molecular weight of human brain expressed SERPINA3 (almost 70 kDa) and chymotrypsin (25 kDa) (Fig. 6a). Incubation of chymotrypsin in brain homogenates from non-neurodegeneration-affected control did not show any ability of SERPINA3 to form a SDS-stable complex together with its target protease.

Similar results were obtained from the incubation of chymotrypsin with brain homogenates from prion-affected mouse brains. In particular, the SerpinA3n-chymotrypsin complex band was more intense in brain sample of end-stage mouse (RML 5 mpi) compared to the brain homogenate of pre-symptomatic one (RML 3 mpi, Fig. 6b).

### Prion Accumulation Changes upon SerpinA3n Modulation

N2a cells were transfected with pcDNA 3.1 encoding for SerpinA3n and an empty pcDNA3.1 (“Empty Vector,” EV). To check *SerpinA3n* overexpression, RNA was extracted and RT-qPCR was performed. N2a Clone 2 was found to be the cell line expressing the highest level of *SerpinA3n* transcript (Online Resource 12a) and was named “N2a-SerpinA3n*.*” WB analysis of N2a-SerpinA3n cell lysate and CM demonstrated the transfection efficacy (Online Resource 12b). To test whether both recombinant and N2a that produced SerpinA3n were able to act as protease inhibitors, a complex formation assay was performed. Molar ratio of 1:5 of chymotrypsin: recombinant SerpinA3n revealed the appearance of a band around 70 kDa corresponding to the sum of the molecular weight of recombinant SerpinA3n (around 47 kDa) and chymotrypsin (25 kDa) (Online Resource 12c). Similarly, N2a-secreted SerpinA3n was able to form a covalent and SDS-resistant complex with chymotrypsin. In this case, the molecular weight of the final complex, around 90 kDa, was relative to the sum of a glycosylated form of SerpinA3n (around 55–60 kDa) and chymotrypsin (Online Resource 12d).

**Fig. 7:**
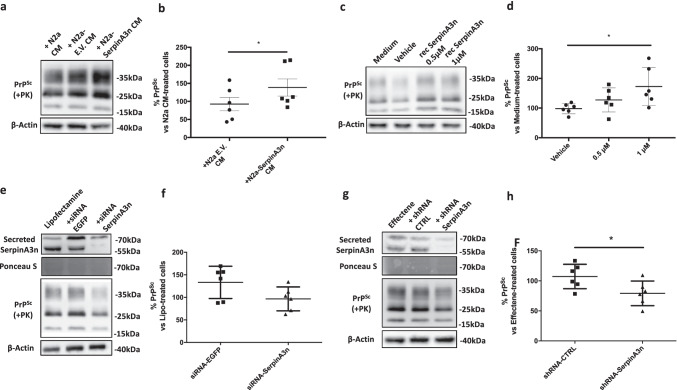
SerpinA3n modulation alters PrP^Sc^ level in ScN2a RML cells. **a**, **c** Representative WB image of PrP^Sc^ in lysates from ScN2a RML treated with CM from N2a, N2a-EV, and N2a-SerpinA3n (**a**) or treated with recombinant SerpinA3n (0.5 µM and 1 µM), vehicle (10 mM Tris–HCl, 50 mM KCl, and pH 8.0), and medium alone (**c**). β-actin was used as protein loading control. PrP^Sc^ signal was developed on another membrane after PK-digestion of cell lysates. **b**, **d** Densitometric analysis of β-actin-normalized PrP^Sc^ levels in N2a-EV and N2a-SerpinA3n CM–treated ScN2a RML relative to cell treated with CM from N2a (**b**, *n* = 6) or in recombinant SerpinA3n and vehicle-treated N2a relative to cell treated with medium only (**d**, *n* = 6). Statistical significance was performed by the Wilcoxon matched pairs signed rank test (**b**) or by the Friedman test with Dunn’s multiple comparisons test (**d**), *p < 0.05. **e**, **g** Representative WB image of PrP^Sc^ in lysates from ScN2a RML transfected with siRNA-SerpinA3n (**e**) and shRNA-SerpinA3n (**g**). β-actin and Ponceau staining were used as protein loading control. PrP^Sc^ signal was developed on another membrane after PK-digestion of cell lysates. **f**, **h** Densitometric analysis of β-actin-normalized PrP^Sc^ levels in siRNA-EGFP and siRNA-SerpinA3n–transfected cells relative to ScN2a RML cells transfected with Lipofectamine only (**f**, *n* = 6) or in shRNA-CTRL and shRNA-Serpina3n–transfected cells relative to ScN2a RML cells transfected with Effectene only (**g**, *n* = 6). Statistical significance was performed by the Wilcoxon matched pairs signed rank test, *p < 0.05

Treating ScN2a RML cells with CM of N2a-SerpinA3n or N2a-EV and N2a cells as controls has tested the SerpinA3n-mediated modulation of prion accumulation. PK-digestion and WB analysis after seventy-two hours treatments of prion-infected cell lysates revealed an average 40% increased prion accumulation in cells treated with the conditioned medium of N2a-SerpinA3n compared to the ones that received N2a EV CM (Fig. 7a, b). To test whether the increased PrP^Sc^ signal could be mediated by a non-glycosylated form of SerpinA3n, ScN2a RML cells were treated with two different concentrations of recombinantly produced SerpinA3n. Both 0.5 µM and 1 µM were responsible for an increased prion accumulation (Fig. 7c); however, only 1 µM SerpinA3n showed a statistically significant increase of PrP^Sc^ compared to vehicle-treated cells (Fig. 7d). Notably, a slight increase in PrP^C^ levels was observed in cells treated with N2a-SerpinA3n CM (Online Resource 13a, b) and 1 µM recombinant SerpinA3n (Online Resource 13c, d).

Since the upregulation of SerpinA3n seems to be associated with an increased prion accumulation, SerpinA3n inhibition has been evaluated to test whether it was able to exert any anti-prion effect. SerpinA3n downregulation was obtained both with siRNA and shRNA techniques, leading to a substantial decrease of secreted SerpinA3n expression levels in transfected ScN2a RML cells (Fig. 7e-h). Seventy-two–hour treatments of RML–infected N2a cell, with either *SerpinA3n*-designed siRNA or shRNA, led to a 20–30% reduction of PrP^Sc^ compared to cell transfected with the respective controls (Fig. 7f, h). In particular, *SerpinA3n*-directed shRNA transfection led to a statistically significant reduction of prion load (Fig. 7h).

## Discussion

To address whether other members of the serpin superfamily, besides the already known prion-related upregulation of SERPINA3 [22], were differentially expressed in prion and prion-like pathology, RT-qPCR analysis was performed on post-mortem human frontal cortex samples.

To understand which serpin superfamily members were expressed in brain tissue, we took advantage of online available RNA-sequencing datasets. Although a gold standard criterion does not exist for the setting of an arbitrary threshold to discriminate the expression of a particular transcript in a given tissue sample, we relied on some studies found in literature. Hebenstreit and collaborators chose a cut-off RPKM value of 1 to discriminate between the presence and absence of a particular transcript [58]. Furthermore, to distinguish between expressed and non-expressed genes in a dataset, TPM ≥ 2 was identified as a correct threshold to consider a gene as highly transcribed [57]. Interestingly, this criterion was also consistent with RPKM value of 1, identified by Hebenstreit and collaborators [58], thus confirming the coherence between the two cut-off values and the selection method set. Therefore, we decided to choose RPKM ≥ 1 or TPM ≥ 2 to select serpins expressed in the brain.

The use of three reference genes for normalization is now a gold standard to reduce experimental errors and tissue-derived effects on RT-qPCR [73, 74]. Thus, we used *GAPDH*, *B2M*, and *ACTB*, displaying comparable expression levels across the analyzed groups [67], as reference genes to consolidate the relative gene expression levels in our brain samples.

Together with the relevant upregulation of *SERPINA3* in sCJD and AD at early stages of NFT pathology frontal cortex, our transcriptomic analysis revealed subtle dysregulations of other *SERPIN* members in neurodegenerative diseases. *SERPINB1* was upregulated in both CJD and AD at early stages of NFT pathology cases. It is a key regulator of neutrophil-programmed cell death, expressed in human brain at the level of microglia and macrophages [75]. In line with our results, genetic variation of *SERPINB1* seems to be associated to amyloidosis in a sex-specific manner [76] and, additionally, its expression in prefrontal cortex has been related to amyloid burden [77], further supporting the possible involvement of *SERPINB1* in neurodegeneration.

Another member of clade B serpins, *SERPINB6*, was upregulated in sCJD patients. This transcript encodes for a widely expressed nucleocytoplasmic serpin, which exerts a protective role against cell death induced by proteases ectopic release or internalization, typical of infection status or cerebral ischemia [78]. It was recently shown that pharmacological inhibition of proteasome in PC12 cells results in α-synuclein inclusion body formation and upregulation of *SERPINB6*, suggesting a role for this serpin in response to cellular insults [79]. In this respect, since ubiquitin proteasome system activity is fundamental in preventing the accumulation of misfolded proteins [80], it is possible that *SERPINB6* dysregulation may also have a function in other neurodegenerative diseases. *SERPINB6* is a known inhibitor of kallikrein-8 [81], a brain-expressed serine-protease involved in hippocampal plasticity [82]. Although further experiments are needed, *SERPINB6* inhibition of synaptic plasticity-related kallikrein-8 activity may contribute to the neurodegeneration process.

The sCJD group revealed an upregulation of *SERPING1*, also known as complement I esterase inhibitor, used as marker of the A1 states of astrocytes [83]. The A1 state of astrocytes can be associated with a loss of astrocyte ability to promote neuronal survival and with the induction of neuronal and oligodendrocyte cell death [84]. Indeed, when the induction of A1 phenotype is prevented, astrocytes assume a neuroprotective role in neurodegeneration [83]. In line with our findings, a significant increase in expression of A1 markers, including *SERPING1*, was observed in cortex and hippocampus of prion-infected mice [83]. Increased expression, and possibly, inhibitory activity of *SERPING1* in sCJD brains could occur in response to complement activation, already associated to prion diseases [85].

*SERPINH1*, upregulated in the frontal cortex from sCJD patients, was proposed as marker of microglial activation since it was upregulated in Aβ-stimulated human microglial cells [86]. Considering the key role of microglia in inflammatory response in neurodegeneration, *SERPINH1* may also act as microglia activator in prion pathology. In addition, *SERPINH1* expression was found to be increased in both AD and PD, indicating that its overexpression may correlate with different neurodegenerative diseases [87].

Among the nine analyzed human *SERPIN* transcripts, only *SERPINI1* resulted to be statistically significant downregulated in sCJD patients. *SERPINI1*, known as neuroserpin, is an inhibitor of trypsin-like serine proteases widely expressed throughout the nervous system [88], which can cause, upon mutation and further intracellular polymer accumulation, a disease named familial encephalopathy with neuroserpin inclusion bodies. This particular form of dementia is responsible for the formation of inclusion bodies in cortical and subcortical neurons, resulting in neuronal degeneration [89]. Controversial evidence concerning the role of *SERPINI1* in AD also exists. Indeed, neuroserpin can bind to Aβ and alter its oligomerization, thus having a neuroprotective role [90] or it may be a detrimental factor by reducing the clearance of Aβ [91]. However, in our analysis, we did not observe any dysregulation in *SERPINI1* expression in the AD group. In light of the aforementioned observations and the role of neuroserpin in neuroprotection and neurodegeneration [88], *SERPINI1* may be involved in prion diseases as well as in other neurodegenerative diseases; whether its involvement is beneficial or detrimental remains to be understood. Furthermore, *SerpinI1* was downregulated in RML–infected mice when normalized to *Gapdh* (Online Resource 7), corroborating the hypothetical role of this serpin in prion pathology.

Given the high sequence homology between human and mouse serpins [12], we wondered about a possible correlation in differentially expressed serpins in human and mouse. For these reasons, we performed transcriptomic analysis in whole brain of RML–infected CD1 mice and huAPP^Swe^/moAPP^0/0^, two mouse models recapitulating prion and AD, respectively [92, 93].

The upregulation of *SerpinA3n* observed in RML–infected CD1 mouse brains [22] was paralleled by an increased expression, although to a lesser extent, of this transcript in huAPP^Swe^/moAPP^0/0^ mice compared to control group. Similarly, WB analysis revealed an increased SerpinA3n protein expression in huAPP^Swe^/moAPP^0/0^ mice compared to moAPP^0/0^ animals. SerpinA3n upregulation in brain of huAPP^Swe^/moAPP^0/0^ mice confirmed the key role played by SerpinA3n in human and mouse neurodegeneration. This upregulation was also found in oligodendrocytes surrounding the Aβ-plaques in another AD mouse model [50], further supporting our results and the correlation between SERPINA3/SerpinA3n and AD.

Interestingly, the mild upregulation of mouse SerpinA3n at both transcript and protein levels in the AD mouse model used in the present study could be associated with the age at which mice were sacrificed. Indeed, the first characterization of APP23 mouse line pointed out a direct association between Aβ plaques and mouse age, supported by an increasing size and number of Aβ deposits with the increasing age of mice [94]. For this reason, only in 2–3-year-old mice are most of the brain regions characterized by Aβ plaques [93]. Considering that huAPP^Swe^/moAPP^0/0^ mice start to develop amyloid deposits after 10 months [95], Aβ-plaques could not be diffused in all mouse brain regions at 12 months of ages. In light of the already suggested direct correlation between Aβ-related pathology and SERPINA3 [49, 50], the mild upregulation of *SerpinA3n* found in huAPP^Swe^/moAPP^0/0^ mice could reflect the low numbers of Aβ-plaques present in our AD mouse model at 12 months of age.

Although *SerpinA3n* resulted to be one of the most upregulated transcripts in prion-infected brain, our analysis revealed a significant upregulation of *SerpinF2* in RML–infected mouse brain. *SerpinF2*, also known as α_2_-antiplasmin, is an inhibitor of plasmin [96]. Plasmin is a serine protease formed upon the proteolytic cleavage of its precursor, plasminogen, by tissue-type plasminogen activator (t-PA) or by the urokinase-type plasminogen activator (u-PA). This system is controlled by several inhibitors such as the direct inhibitor of plasmin, *SerpinF2*, or the plasminogen activator inhibitor, *SerpinI1* [97]. Of note, all the components of plasmin cascade are present in the CNS and, although this system is traditionally studied for fibrinolysis, their involvement in neurodegeneration, particularly in AD, has been reported [96, 98]. However, few studies have explored the putative role of *SerpinF2* in prion diseases. Interestingly, both PrP^C^ and PrP^Sc^ can interact with t-PA and plasminogen and can stimulate plasmin formation [99]. Furthermore, several *in vitro* studies have reported that interaction between PrP^Sc^ and plasminogen may favor PrP^Sc^ replication and propagation [100] and that t-PA displays higher expression and activity in mouse models of prion disease [101]. Despite the different hypotheses regarding the correlation between members of the plasmin cascade and prion diseases, none highlights a role for *SerpinF2*. The increased expression of *SerpinF2*, observed in the prion mouse model utilized in the present study, could represent a cellular response to mitigate the strong effects of plasmin activity. Interestingly, the plasmin system may also contribute to the degradation of extracellular protein aggregates, suggesting a possible involvement in neurodegeneration [102]. Therefore, direct inhibition of plasmin by *SerpinF2* may counteract this process, favoring the accumulation of misfolded proteins. On the other hand, the prion-specific decreased expression of *SERPINI1*/*SerpinI1*, a known t-PA and u-PA inhibitor [103], could be fundamental to support the increased fibrinolytic activity possibly required for degradation of PrP^Sc^ plaques.

The most intriguing aspect of our results is that, among the seventeen analyzed serpin transcripts in the brain of prion and AD mouse models, *SerpinA3n* was the only one to be differentially upregulated in mouse brain.

Considering human and mouse serpin homologies, only *SERPINA3* and *SerpinA3n* are differentially expressed in both species, respectively. Conversely, the human orthologue of *SerpinF2*, *SERPINF2*, was not even included in our analysis because of its weak expression in the brain under physiological condition. However, it may be possible that *SERPINF2* expression could be induced only during pathological conditions [104]. Recently, it has been shown that another member of the serpin superfamily, *SERPINA1*, is not expressed at high levels in normal brain tissue [87], but it is overexpressed in AD, frontotemporal lobar degeneration, and CJD, suggesting that its expression may be detrimental for neuronal function [105]. For this reason, differential expression of *SERPINF2* in prion-affected human brain cannot be excluded, even if further work is required to verify this hypothesis.

Overall, our results have shown that several members of the serpin superfamily were dysregulated in prion and prion-like pathology, even if their implication in neurodegeneration is far from being completely elucidated. However, only *SERPINA3*/*SerpinA3n* was markedly upregulated both in human prion and AD–affected patients and in mouse models of these diseases, highlighting its putative role in neurodegeneration.

To investigate whether SERPINA3/SerpinA3n transcript and protein levels are paralleled by its increased biochemical activity, we evaluated SERPINA3 anti-protease activity in frontal cortex samples of AD at early stages of NFT pathology and the activity of SerpinA3n in brain homogenates of prion-infected mice. Incubation with one of its target proteases (chymotrypsin [13]), followed by WB analysis, revealed an increased SERPINA3/SerpinA3n inhibitory activity in both AD and prion-affected samples compared to relative controls. These biochemical evaluations suggested that SERPINA3/SerpinA3n upregulation can occur as a response to the augmented protease activity, which arises to counteract neurodegenerative disease–related protein aggregate accumulation [33].

In order to better investigate SERPINA3/SerpinA3n role in neurodegeneration, we performed *in vitro* experiments to test prion accumulation changes upon SerpinA3n modulation. To test the hypothesis according to which increased SerpinA3n*n* levels would lead to a further accumulation of prions, either N2a-SerpinA3n conditioned medium or recombinant SerpinA3n treatment has been performed. Firstly, the activity of N2a-secreted and recombinant SerpinA3n has been tested using chymotrypsin. Both recombinant and glycosylated, N2a-derived, SerpinA3n demonstrated their ability to form an SDS-stable complex with their cognate proteases resulting in the appearance of a band corresponding to the sum of SerpinA3n (47 kDa, for recombinant and around 55 kDa, for N2a overexpressing SerpinA3n CM) and chymotrypsin (25 kDa) molecular weight (Online Resource 12c, d). ScN2a RML treatment with CM from N2a cells overexpressing SerpinA3n showed an increased prion accumulation compared to CM from empty-vector carrying N2a cell treatment. Similarly, 1 µM recombinant SerpinA3n was responsible for an increased accumulation of prion in ScN2a RML cells compared to the vehicle-treated ones. To test whether the SerpinA3n effect on prion accumulation was limited to PrP^Sc^, N2a-SerpinA3n CM and recombinant SerpinA3n treatment on non-infected N2a cells was performed. Only a trend of PrP^C^ increase was visible in cells treated either with N2a-SerpinA3n CM (Online Resource 13a, b) or with 1 µM recombinant SerpinA3n (Online Resource 13c, d). These results probably indicate a partial SerpinA3n-mediated inhibition of physiological protease-dependent PrP^C^ cleavage, which could be responsible for an increase availability of substrate needed for the pathological conversion into PrP^Sc^.

To further assess the SerpinA3n-prion relationship, the effects of *SerpinA3n* transcriptional inhibition upon PrP^Sc^ were evaluated. Either *SerpinA3n*-targeted siRNA or shRNA transfection led to a reduction of prions compared to control cells. Although *SerpinA3n*-directed siRNA transfection was responsible for a strong, even if not statistically significant, reduction of PrP^Sc^ compared to siRNA-EGFP transfection, only a 5% average reduction compared to Lipofectamine-treated cells has been observed (Fig. 7f), suggesting that siRNA-mediated *SerpinA3n* reduction was not sufficient to reduce prion accumulation. The lack of consistency in prion load reduction between the two transfection methods could be explained by a higher potency, and sustainable effects, characterizing shRNA transfection compared to the siRNA one [106]. These data suggested that modulation of *SerpinA3n* could have an effect on prion accumulation. According to our hypothesis, increased SerpinA3n levels (obtained through N2a-SerpinA3n CM or recombinant SerpinA3n treatment) would lead to increase inhibition of protease(s) responsible for prion clearance, leading to a further pathological accumulation. Conversely, *SerpinA3n* reduction (i.e., at transcriptional levels) will free protease(s) ability to act upon and degrade pathological PrP^Sc^ aggregates.

These results pave a way to better investigate SERPINA3/SerpinA3n contributions to neurodegeneration, for example, through the inhibition of protease-dependent protein aggregate degradation.

Interestingly, the majority of prion and non-prion neurodegenerative disorders manifest symptoms in adulthood. This implies that the amount of pathological proteins accumulating in the brain has to exceed a threshold for pathological processes to be unabated [36, 107]. Thus, probably, the clearance machine would be saturated during aging by an excess of protein aggregates, or the age-dependent increased expression of protease inhibitors, such as SERPINA3 [19], could reduce the protease(s)-dependent clearance mechanism.

## Conclusion

Considering both our current findings and previous studies, we speculate that differential expression of *SERPINA3*/*SerpinA3n*, and to a larger extent of many other serpins, could be shared among neurodegenerative diseases. Despite their involvement in neurodegeneration, the role of serpins still remains elusive.

Concerning humans, *SERPINA3*, *SERPINB1*, *SERPINB6*, *SERPING1*, *SERPINH1*, and *SERPINI1* were dysregulated in sCJD patients, whereas only *SERPINA3* and *SERPINB1* members were differentially expressed in patients at early stages of AD–related pathology. Therefore, *SERPINA3* and *SERPINB1* transcripts were upregulated in both disease-affected groups compared to relative controls, even if *SERPINA3* showed one order of magnitude higher fold change upregulation compared to all the other serpin superfamily members.

In mouse, *SerpinA3n*, together with *SerpinF2*, was differentially expressed in RML–infected CD1 compared to controls. Interestingly, SerpinA3n increases protein level paralleled upregulation of its transcript in brain of AD mouse model.

Future studies are needed to clarify the role of differentially expressed serpins in neurodegenerative disorders; additional investigations of serpin gene expression in other human brain areas, rather than only in the frontal cortex, may also provide a more detailed screening of differentially expressed serpin genes. Eventually, further investigations to address whether the changes found at a transcript level are paralleled by a variation at protein level should be performed, corroborating the possible role of differential serpin gene expression in neurodegenerative processes.

However, among all the members of the serpin superfamily, SERPINA3/SerpinA3n was the only gene upregulated in prion disease and AD in both human samples and mouse models.

The SERPINA3/SerpinA3n transcript and protein level increase was paralleled by its peculiar anti-protease activity in AD at early stages of NFT pathology and prion-infected brain tissues, suggesting a functional reaction to protease activation during the neurodegenerative process.

Moreover, our *in vitro* experiments demonstrated a possible SerpinA3n-dependent prion accumulation. According to the most intriguing hypothesis, it is likely that SERPINA3/SerpinA3n overexpression may favor prion accumulation through the inhibition of proteases probably involved in PrP^Sc^ degradation, while SERPINA3/SerpinA3n inhibition may be responsible for a reduction in prion load.

SERPINA3/SerpinA3n marked dysregulation in prion and Alzheimer’s diseases, and its effect on the prion accumulation process, suggests its consideration as a potential therapeutic target. Further analyses in other neurodegenerative disorders are needed to understand whether the neurodegenerative mechanism is SERPINA3/SerpinA3n-dependent or whether other serpin superfamily members are involved in these pathological processes.

## Supplementary Information

Below is the link to the electronic supplementary material.

Supplementary file1 (PDF 3274 KB)

## Data Availability

All data generated or analyzed during this study are included in this published article (and its supplementary information file).
