# Supplementary material for: Serpin Signatures in Prion and Alzheimer’s Diseases
Source: Mol Neurobiol. 2022 Apr 13;59(6):3778–99. doi: 10.1007/s12035-022-02817-3 (PMC9148297; doi:10.1007/s12035-022-02817-3)
Supplement: Supplementary file 1 — Supplementary file1 (PDF 3274 KB) [file 12035_2022_2817_MOESM1_ESM.pdf]

Supplementary Information

Manuscript title:

Serpin signatures in Prion and Alzheimer's diseases

Journal Name:

Molecular Neurobiology

Authors:

Marco Zattoni, Marika Mearelli, Silvia Vanni, Arianna Colini Baldeschi, Thanh Hoa Tran, Chiara Ferracin, Marcella Catania, Fabio Moda, Giuseppe Di Fede, Giorgio Giaccone, Fabrizio Tagliavini, Gianluigi Zanusso, James W. Ironside, Isidre Ferrer and Giuseppe Legname

Affiliations and email address of the Corresponding author:

Professor Giuseppe Legname

Laboratory of Prion Biology, Department of Neuroscience, Scuola Internazionale Superiore di Studi Avanzati (SISSA), Trieste, Italy.

legname@sissa.it

Gene	Forward primer sequence 5'→3'	Reverse primer sequence 5'→3'
GAPDH	CCTGCACCACCAACTGCTTA	TCTTCTGGGTGGCAGTGATG
B2M	AGATGAGTATGCCTGCCGTG	TCATCCAATCCAAATGCGGC
RPL19	CTAGTGTCCTCCGCTGTGG	AAGGTGTTTTTCCGGCATC
ACTB	AGAGCTACGAGCTGCCTGAC	AGCACTGTGTTGGCGTACAG
SERPINA3	TGCCAGCGCACTCTTCATC	TGTCGTTCAGGTTATAGTCCCTC
SERPINA8	CCAGCCTCACTATGCCTCTG	GTGGATGGTCCGGGGAGATA
SERPINB1	GGCGGCCTGTCGGTTTT	AGCCGGATTGTTCTCACTCA
SERPINB6	CCCTCCCGCGGTTTAAACTA	CCTCCGTGCCTTCCTCAT
SERPINB8	CGGATGAGGTACACACCCAG	TCCATCACTCTTTCACGGCG
SERPINB9	TCAACACCTGGGTCTCAAAAAA	CAGCCTGGTTTCTGCATCAA
SERPINE1	GCAACGTGGTTTTCTCACCC	GGCCATGCCCTTGTCATCAA
SERPINE2	CGAGCGCGGTCGTCCT	CCCGTGTTGGAGCCTAGTTC
SERPINF1	CCCGTGTTGGAGCCTAGTTC	AACTTTGTTACCCACTGCCCC
SERPING1	CCCATGATGAATAGCAAGAAGTACC	CTGCCCCACCTTGGCTT
SERPINH1	CCGTGGCTTCATGGTGACTCGG	AGTAGTTGTAGAGGCCTGTCCGGT
SERPINI1	AGAGACGAAAGCAGGAACGA	TGTAACAGTTTCAAGCCTCCCA

Online Resource 1 Human reference and target gene primer sequences

Forward (5'-3') and reverse (3'-5') primers of each human reference gene and *SERPINs* transcript analyzed in the present study.

Gene	Forward primer sequence 5'→3'	Reverse primer sequence 5'→3'
Gapdh	TTCACCACCATGGAGAAGGC	GGCATGGACTGTGGTCATGA
Tubb3	CGCCTTTGGACACCTATTC	TACTCCTCACGCACCTTG
ActB	CACACCCGCCACCAGTTC	CACACCCGCCACCAGTTC
SerpinA3n	ACCCTGAGGAAGTGGAAGAA	CCTGATGCCCAGCTTTGAAA
SerpinA6	GCTGGCAGATGTGGGCATTA	TGGCCTTGTGGAGTACCGTT
SerpinA8	TGTCTAGGTTGGCGCTGAAG	GATGTATACGCGGTCCCCAG
SerpinB1a	GTTTTCCTCCTCGGCTTTTGC	AGTTTGAGGATGGAGTCGTCC
SerpinB1b	GTGCTTGCCAGTAAGACACTC	ATGGTGAAGGCTCCTCTGTAG
SerpinB6a	TTCCTGCACCCTTCTGTGTC	TGAAGCCGCCTAGATTCTCC
SerpinB8	TCGTGTGATTTCCTTTCGACCT	TTCCTGCACCCTTCTGTGTC
SerpinB9d	AAAGAACCCATGGAACGGGA	TCCTGAGCCTGAGAGCTTAC
SerpinD1	GAATGGCAATATGTCAGGCATCT	CACTGTGATGGTACTTTGGTGCTT
SerpinE1	TCCACAAGTCTGATGGCAGC	GGGGTGGTGAACTCAGTGTA
SerpinE2	CACATGGGATCGCGTCCATC	CAGCACTTTACCAACTCCGTTTA
SerpinE3	TGGAGCTTTCAGAGGAGGGTA	TACTGAAGACAAACCCTGTGCT
SerpinF1	ACGATCTGTACCGCCTGAGA	TTCGATGTTCAGCTCCCAGAG
SerpinF2	TTCTCCTCAACGCCATCCA	GGTGAGGCTCGGGTCAAAC
SerpinG1	GAACTTGGACCAGGACGCAG	GCTGGTAGCTTCGGGATCTG
SerpinH1	CCGGCCCAGAATGAAAAAGGC	TAAGGTGCCCAGAAGGAGAGA
SerpinI1	CGCCATTCAATGGGATATG	CAAAGAGCGAATTGGCAAG

Online Resource 2 Murine reference and target gene primer sequences

Forward (5'-3') and reverse (3'-5') primers of each murine reference gene and *Serpins* transcript analyzed in the present study.

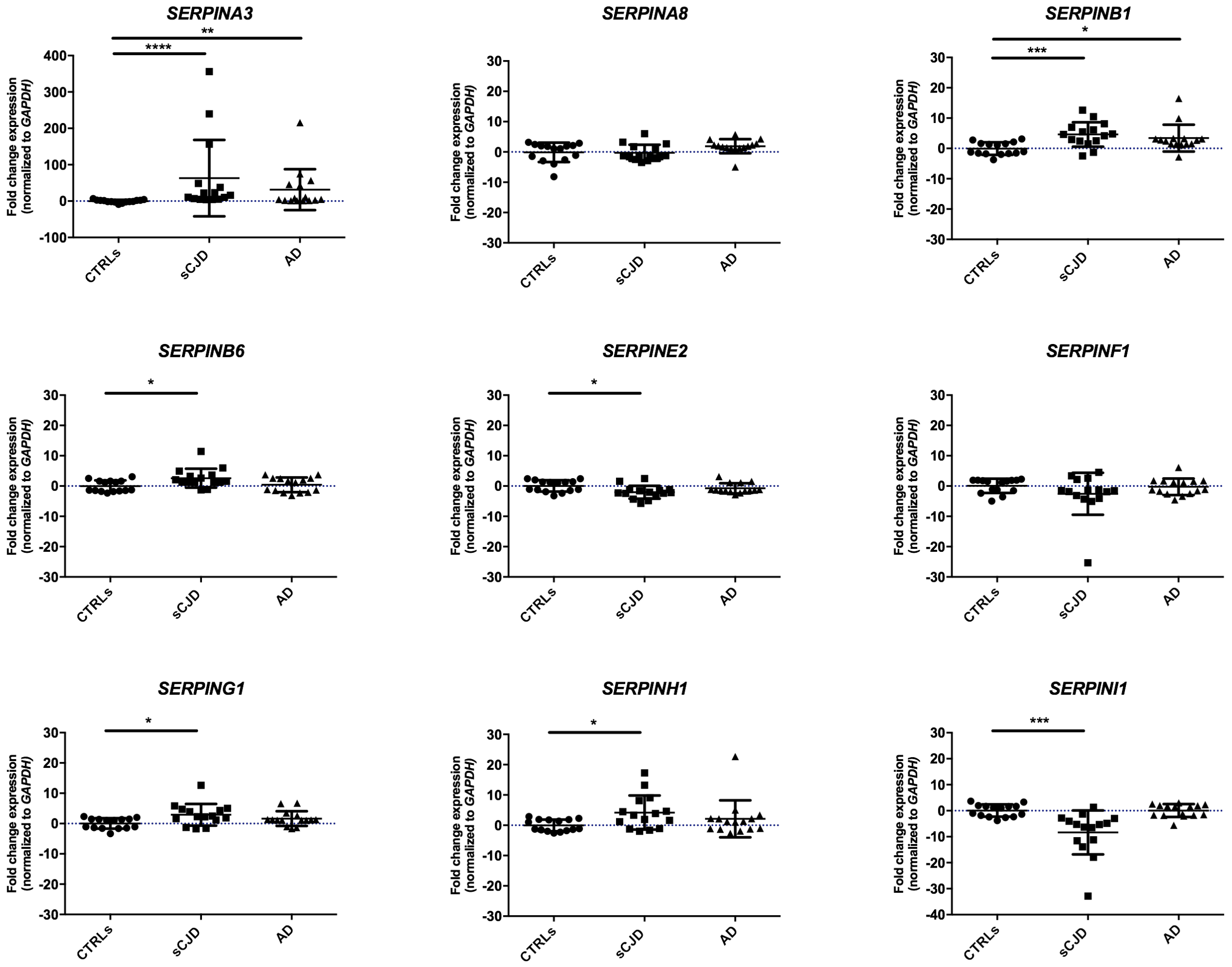

Online Resource 3 *SERPINs* expression level in sCJD and AD human brain samples normalized to *GAPDH* RT-qPCR for *SERPINs* mRNA expression in sCJD (n=15) and AD (n=15) relative age-matched controls (CTRLs, n=15) frontal cortex samples normalized to *GAPDH* as reference gene. Statistical analysis was performed using the Kruskal-Wallis test with Dunn's multiple comparisons test. Adjusted *p* value *<0.05, **<0.01, ***<0.001, **** <0.0001.

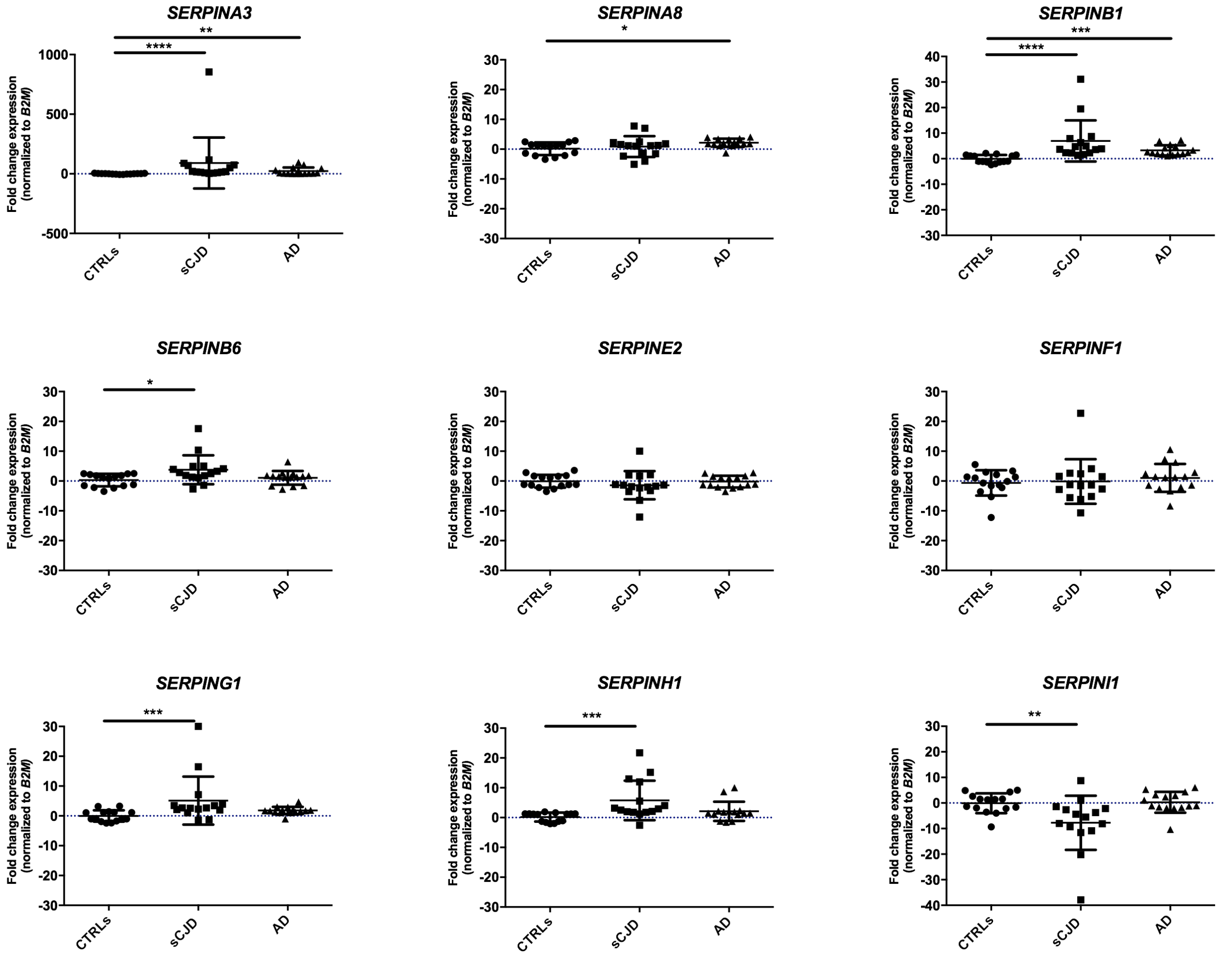

Online Resource 4 *SERPINs* expression level in sCJD and AD human brain samples normalized to *B2M*

RT-qPCR for *SERPINs* mRNA expression in sCJD (n=15) and AD (n=15) relative age-matched controls (CTRLs, n=15) frontal cortex samples normalized to *B2M* as reference gene. Statistical analysis was performed using the Kruskal-Wallis test with Dunn's multiple comparisons test. Adjusted *p* value *<0.05, **<0.01, ***<0.001, **** <0.0001.

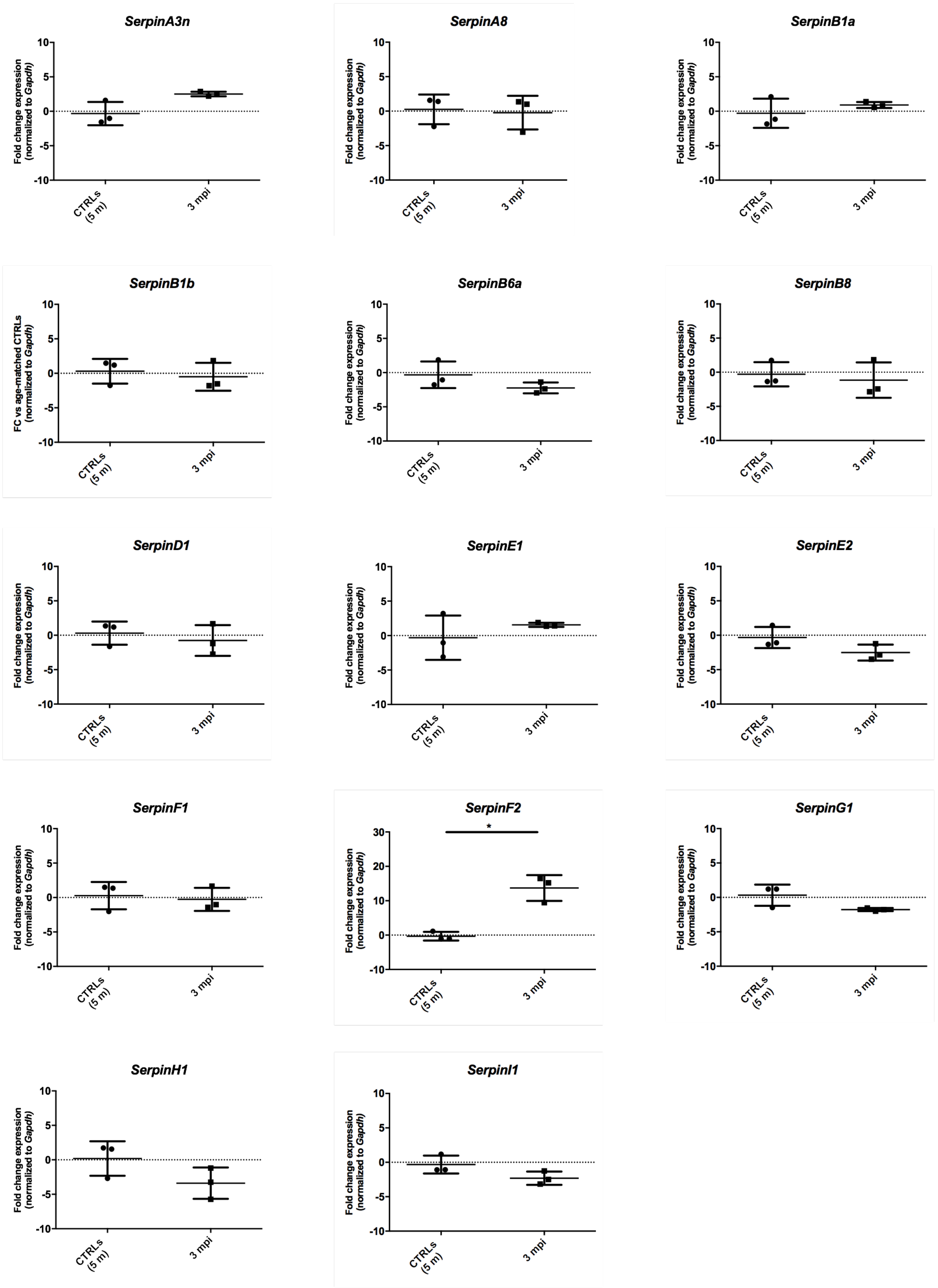

Online Resource 5 ***Serpins*** **expression level in pre-symptomatic RML-infected CD1 mouse brain normalized to** ***Gapdh***

RT-qPCR analysis for *Serpins* mRNA expression in 3 months post infection (3 mpi, n=3) and relative age-matched controls whole brain samples (CTRLs 5 m, n=3) normalized to *Gapdh* as reference gene. Statistical analysis was performed with the Mann-Whitney test. Adjusted *p* value * <0.05.

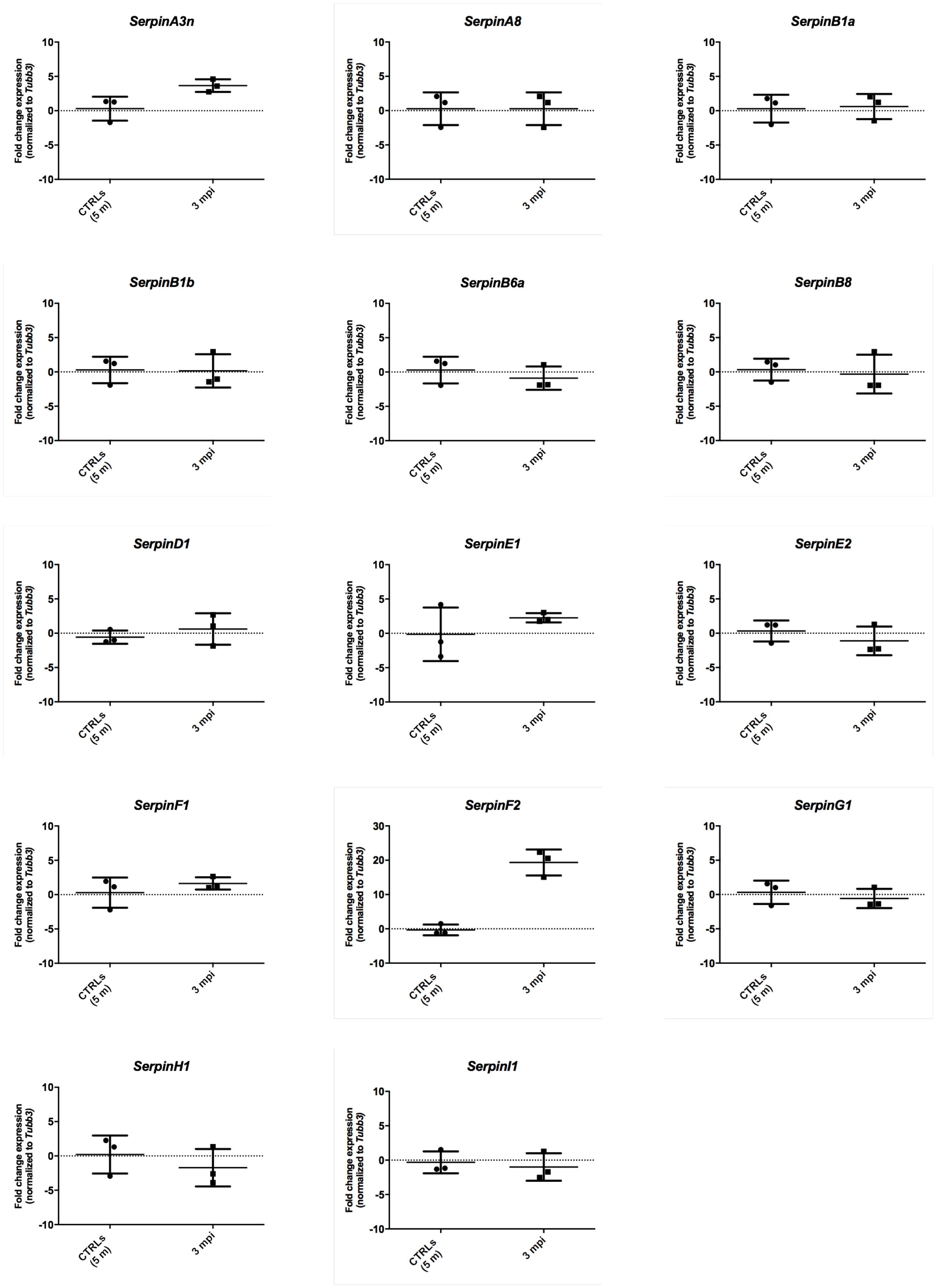

Online Resource 6 ***Serpins*** **expression level in pre-symptomatic RML-infected CD1 mouse brain normalized to** ***Tubb3***

RT-qPCR analysis for *Serpins* mRNA expression in 3 months post infection (3 mpi, n=3) and relative age-matched controls whole brain samples (CTRLs 5 m, n=3) normalized to *Tubb3* as reference gene. Statistical analysis was performed with the Mann-Whitney test.

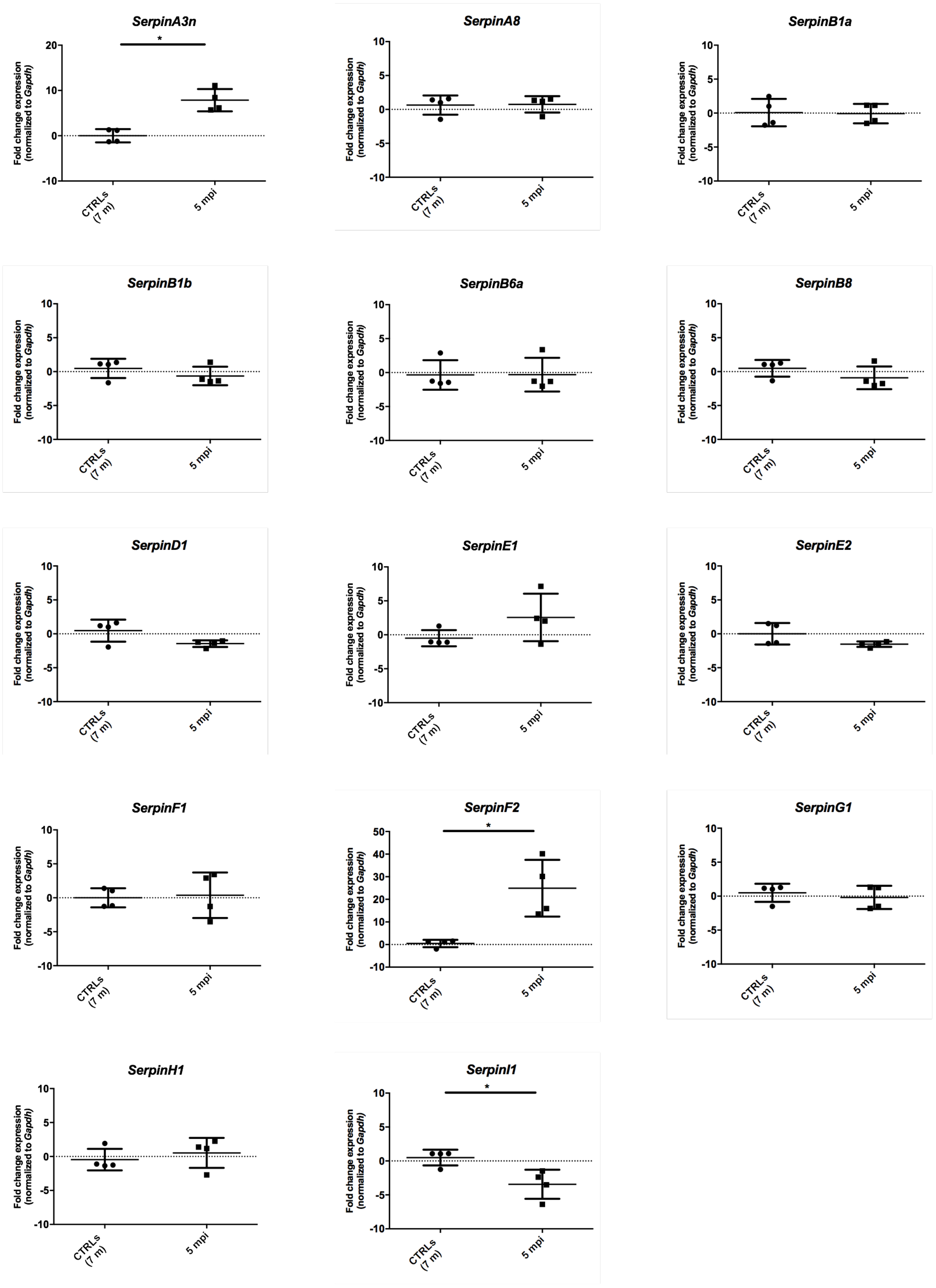

Online Resource 7 ***Serpins*** **expression level in symptomatic RML-infected CD1 mouse brain normalized to** ***Gapdh***
RT-qPCR analysis for *Serpins* mRNA expression in 3 months post infection (5 mpi, n=4) and relative age-matched controls whole brain samples (CTRLs 7 m, n=4) normalized to *Gapdh* as reference gene. Statistical analysis was performed with the Mann-Whitney test. Adjusted *p* value * <0.05.

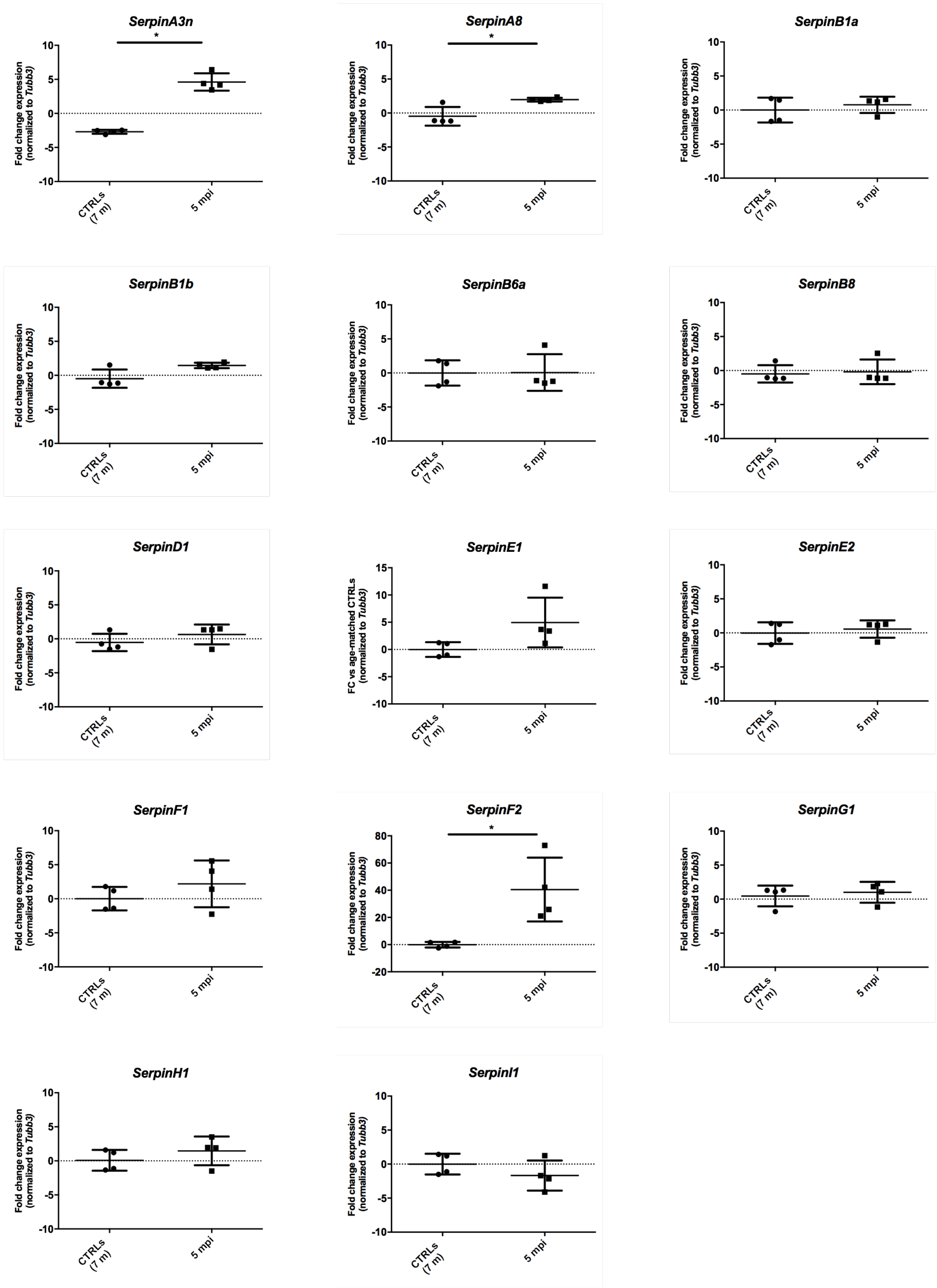

Online Resource 8 ***Serpins*** **expression level in symptomatic RML-infected CD1 mouse brain normalized to** ***Tubb3***

RT-qPCR analysis for *Serpins* mRNA expression in 3 months post infection (5 mpi, n=4) and relative age-matched controls whole brain samples (CTRLs 7 m, n=4) normalized to *Tubb3* as reference gene. Statistical analysis was performed with the Mann-Whitney test. Adjusted *p* value * <0.05.

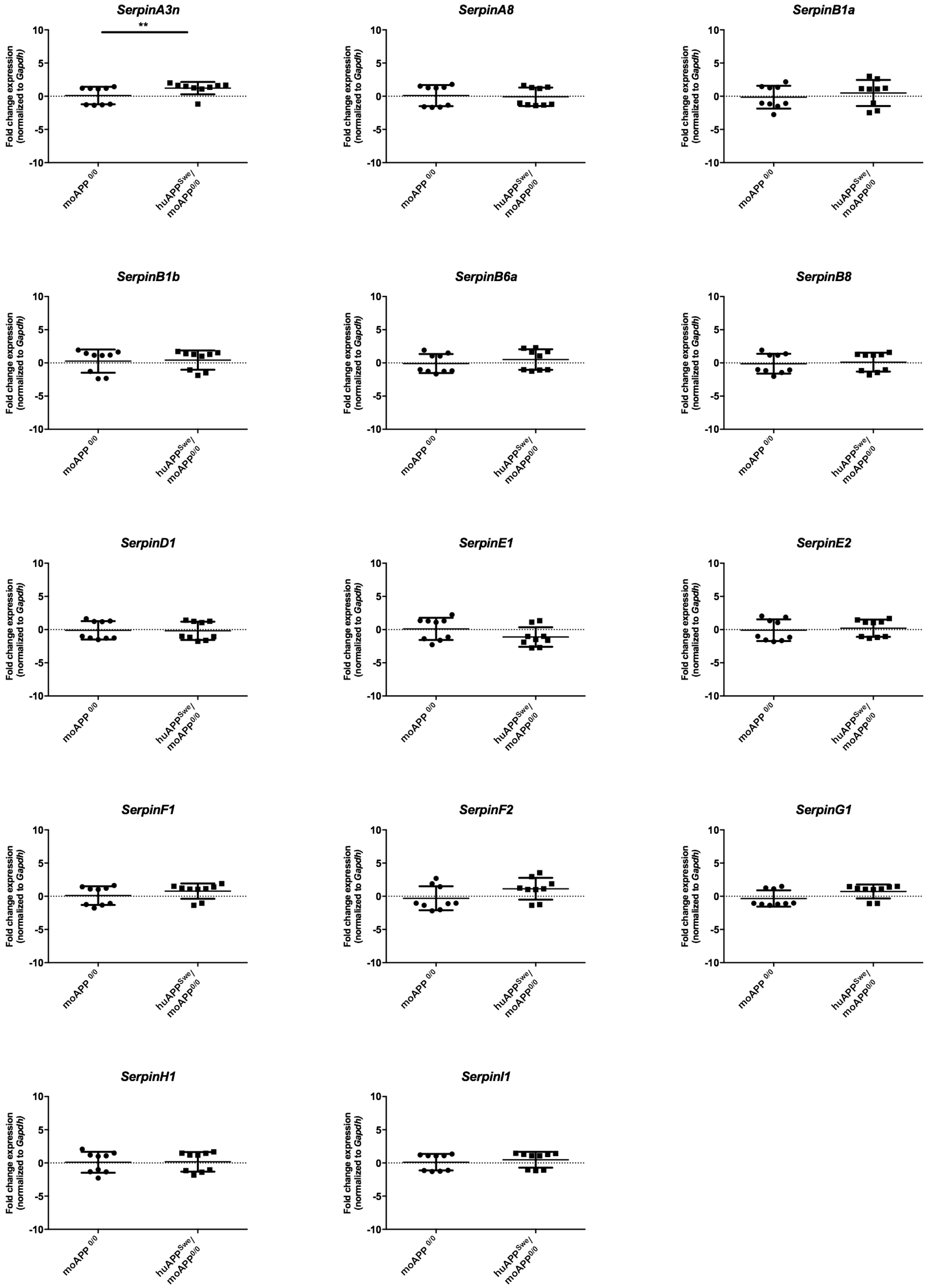

Online Resource 9 *Serpins* expression level in huAPP^{Swe}/moAPP$^{0/0}$ mouse brain normalized to *Gapdh*

RT-qPCR analysis for *Serpins* mRNA expression in huAPP^{Swe}/moAPP$^{0/0}$ (n=9) and relative age-matched controls whole brain samples (moAPP$^{0/0}$, n=9) normalized to *Gapdh* as reference gene. Statistical analysis was performed with the Mann-Whitney test. Adjusted p value **<0.01.

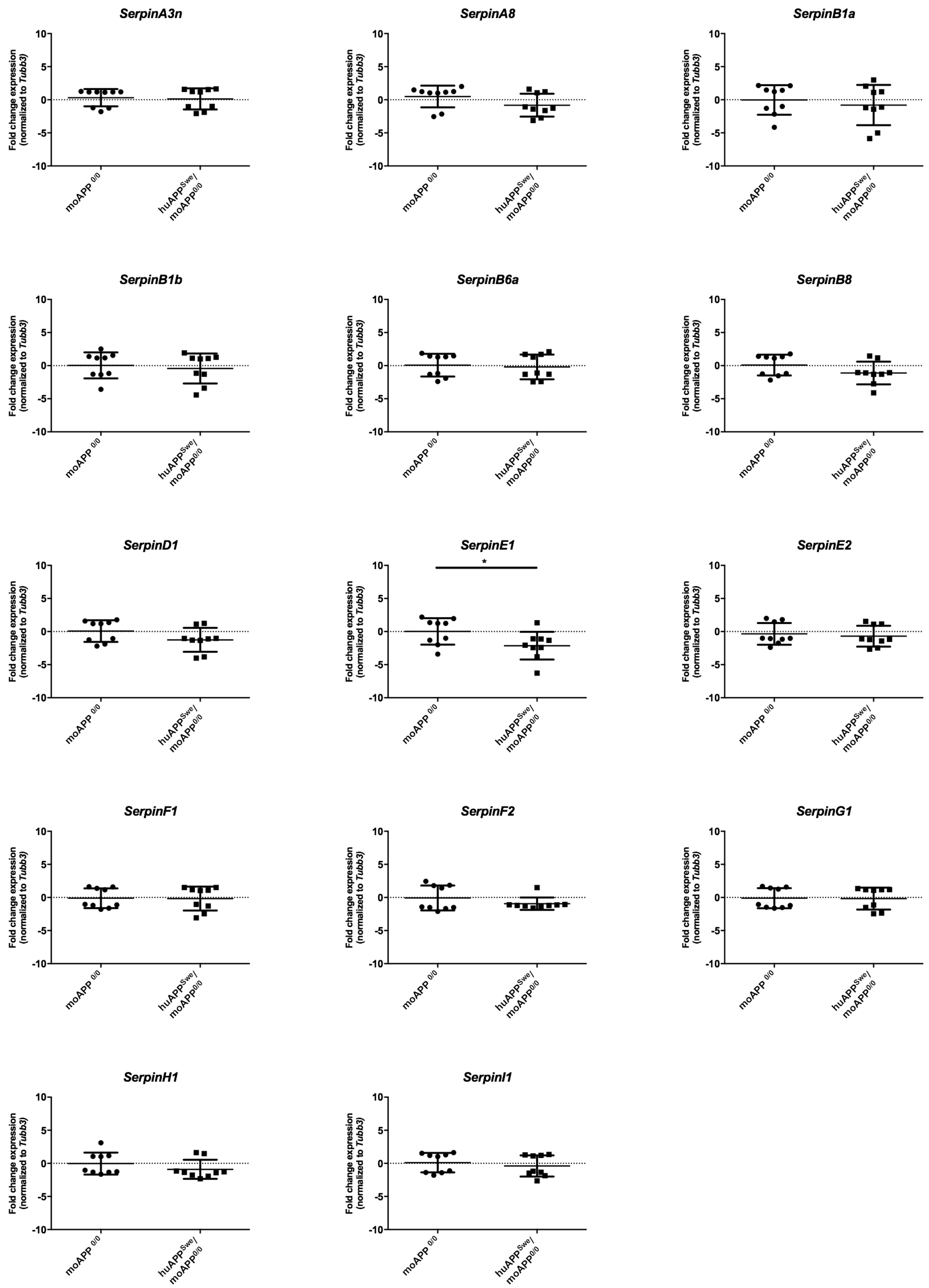

Online Resource 10 ***Serpins*** **expression level in huAPP^{Swe}/moAPP$^{0/0}$ mouse brain normalized to** ***Tubb3***

RT-qPCR analysis for *Serpins* mRNA expression in huAPP^{Swe}/moAPP$^{0/0}$ (n=9) and relative age-matched controls whole brain samples (moAPP$^{0/0}$, n=9) normalized to *Tubb3* as reference gene. Statistical analysis was performed with the Mann-Whitney test. Adjusted *p* value *<0.05.

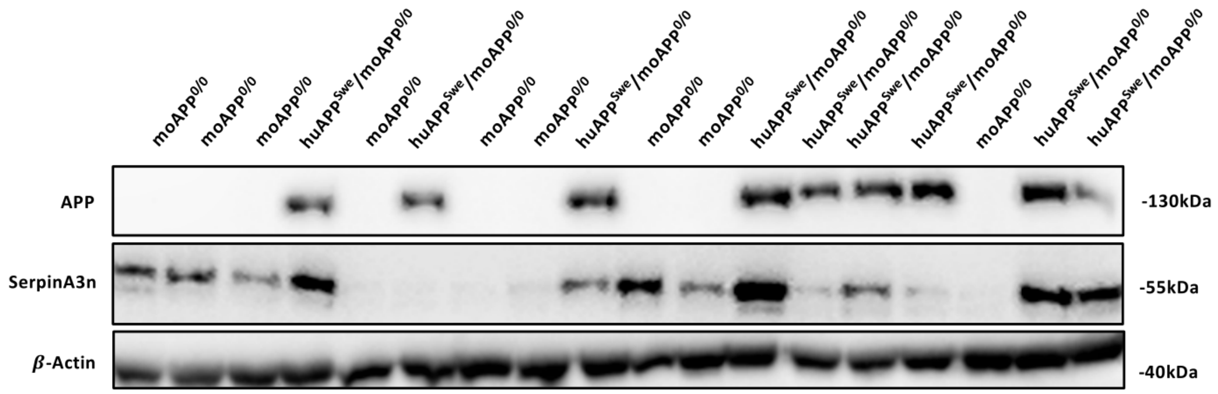

Online Resource 11 Western blotting analysis for SerpinA3n and APP in the brain of moAPP$^{0/0}$ and huAPP^{Swe}/moAPP$^{0/0}$ mouse

WB image of SerpinA3n and APP protein level in moAPP$^{0/0}$ and huAPP^{Swe}/moAPP$^{0/0}$ mouse brain lysates. β-actin was used as proteins loading control and to normalize the expression level of SerpinA3n for densitometric analysis.

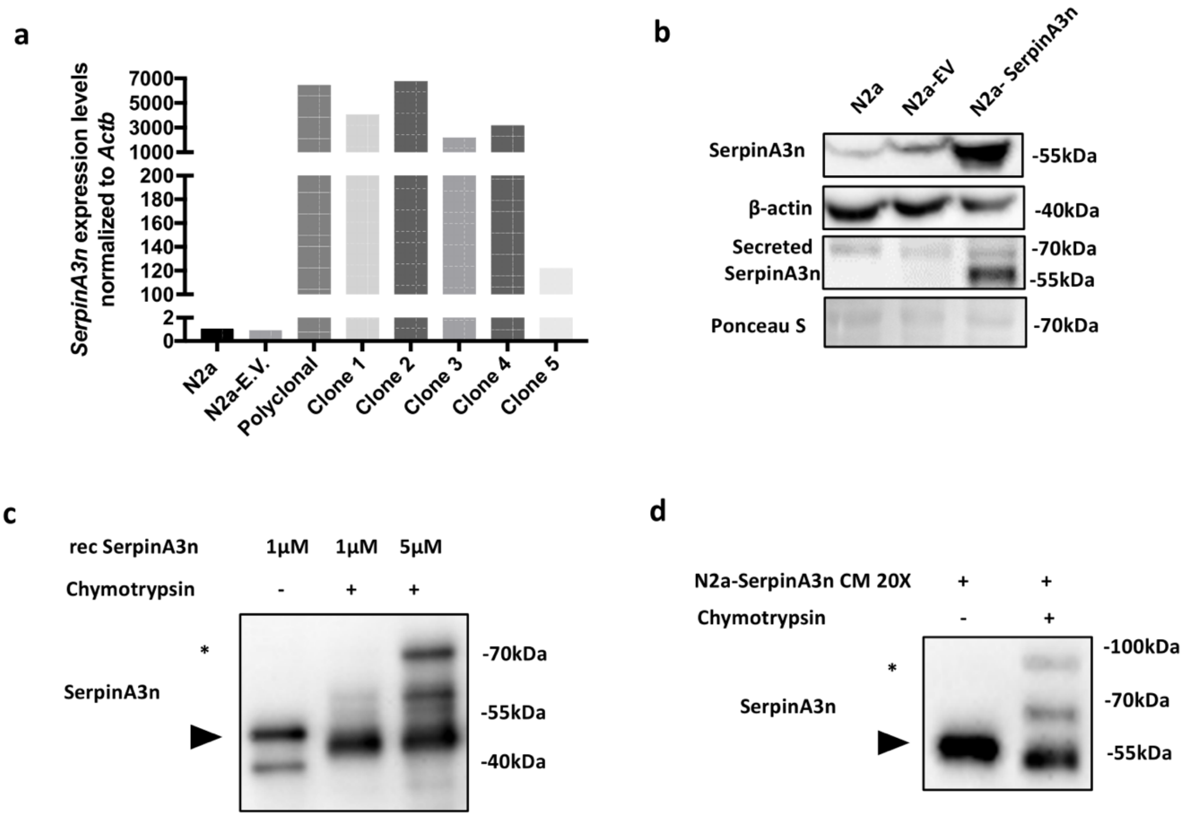

Online Resource 12 Characterization of and N2a-secreted and recombinant SerpinA3n

a Gene expression analysis of SerpinA3n N2a cells compared to not transfected N2a, normalized to *Actb* as reference gene. **b** Representative WB image of intracellular and secreted SerpinA3n on transfected (N2a-EV and N2a-SerpinA3n) and control N2a cells. β-actin and Ponceau Staining were used as proteins loading control. **c, d** Representative WB images of 1 μM chymotrypsin incubated with 1 μM and 5 μM recombinant SerpinA3n or 10 mM Tris-HCl, 50 mM KCl, pH 8.0 (**c**) or CM from N2a-SerpinA3n 20X concentrated incubated with 100 ng of chymotrypsin or medium (**d**). Black arrowhead indicates recombinant SerpinA3n (around 47 kDa, **c**) or the secreted and glycosylated SerpinA3n (55-60kDa, **d**), while asterisk indicates the SDS-resistant covalent complex band at 70 kDa (**c**) or at 90 kDa (**d**) corresponding to the sum of the molecular weight of SerpinA3n and the cognate protease (25 kDa).

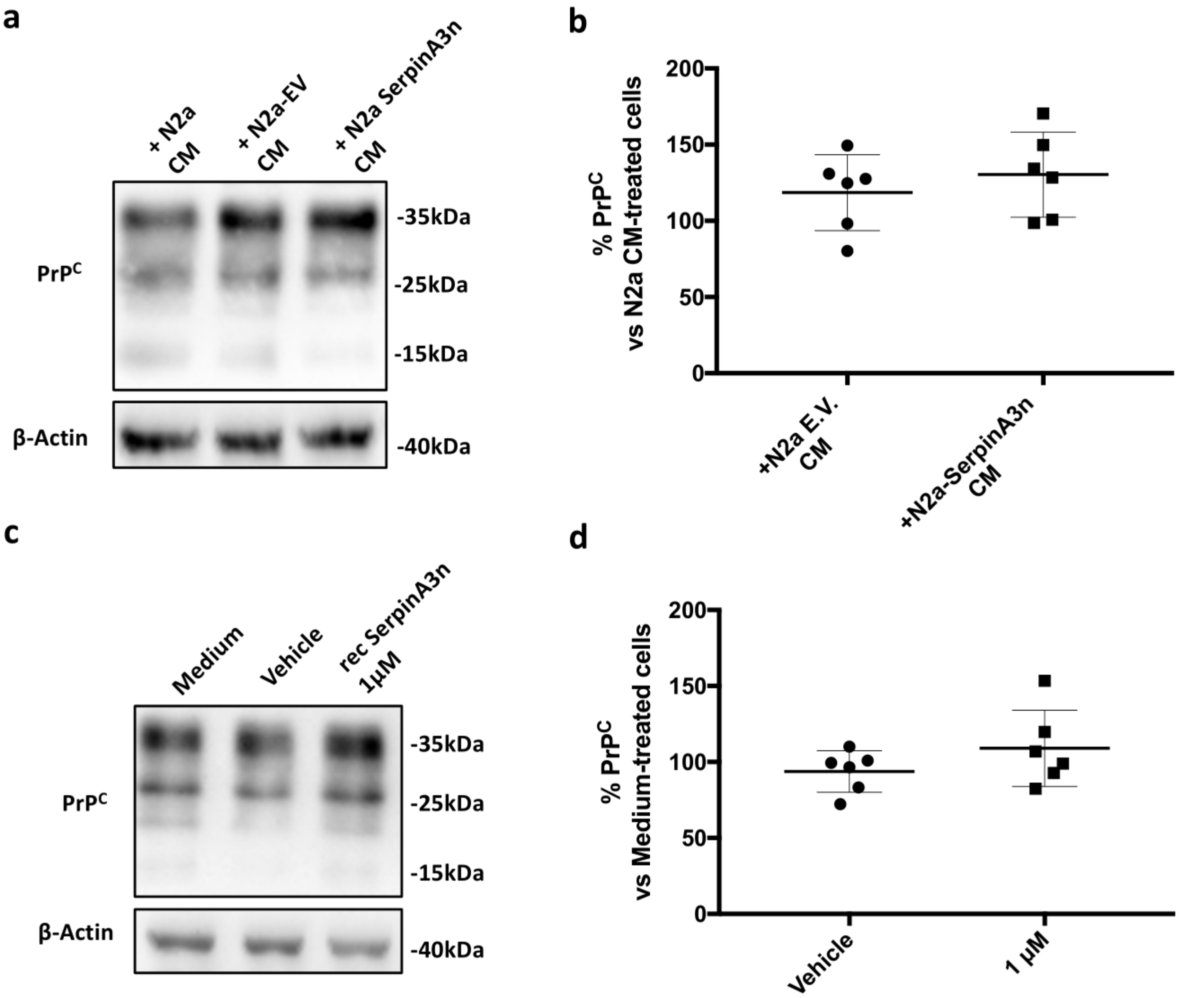

Online Resource 13 PrP^{C} levels in recombinant and N2a-produced SerpinA3n in N2a cells

a, c Representative WB image of PrP^{C} in lysates from N2a treated with CM from N2a, N2a-EV and N2a-SerpinA3n (**a**) or treated with recombinant SerpinA3n (1µM), vehicle (10 mM Tris-HCl, 50 mM KCl, pH 8.0) and medium alone (**c**). β-actin was used as protein loading control. **b, d** Densitometric analysis of β-actin-normalized PrP^{C} levels in N2a-EV and N2a-SerpinA3n CM-treated N2a relative to cell treated with CM from N2a (**b**, n=6) or in recombinant SerpinA3n and vehicle-treated N2a relative to cell treated with medium only (**d**, n=6). Statistical significance was performed by the Wilcoxon matched pairs signed rank test, $^{*}p < 0.05$.
